# Diversity but Not Overall Abundance of Moths and Butterflies (Insecta: Lepidoptera) Decreases around Two Arctic Polluters

**DOI:** 10.3390/insects13121124

**Published:** 2022-12-05

**Authors:** Mikhail V. Kozlov, Vitali Zverev, Elena L. Zvereva

**Affiliations:** Department of Biology, University of Turku, FI-20014 Turku, Finland

**Keywords:** industrial pollution, sulphur dioxide, heavy metals, forest, tundra, Murmansk region

## Abstract

**Simple Summary:**

Biodiversity loss is one of the biggest challenges faced by humankind. Alarming reports on the rapid decline of insect populations call for the exploration of potential drivers of this process. Here, we test the hypothesis that decreases in the abundance and diversity of insects can be caused by industrial pollution. We found that the diversity of moths and butterflies declined in the severely degraded habitats (termed industrial barrens) adjacent to two metallurgical enterprises located in a polar region, but did not respond to moderate levels of sulphur dioxide and heavy metal pollution. Surprisingly, the overall abundance of these insects was not affected even by the extreme pollution loads. This pattern resulted from idiosyncratic responses of moth and butterfly species to pollution, which vary from significantly negative to significantly positive. The patterns in diversity and abundance do not differ between the areas affected by the two studied enterprises, and they are consistent with patterns previously found near another non-ferrous smelter. We conclude that arctic communities of moths and butterflies are unexpectedly tolerant to industrial pollution.

**Abstract:**

Alarming reports on the rapid decline of insects during the past decades call for the exploration of potential drivers of this process. Here, we test the hypothesis that the overall abundance and diversity of moths and butterflies (Lepidoptera) decrease under the impact of industrial pollution in the fragile arctic environment. For this purpose, experienced collectors netted adult Lepidoptera at five tundra sites located 0.5 to 45.3 km from the ore-roasting plant in Zapolyarnyy and at five forest sites located 1.4 to 37.8 km from the copper–nickel smelter at Nikel, in the Murmansk region of Russia. The analysis of the 100 samples collected from 2003 to 2008 and containing 2312 individuals of 122 species revealed that the diversity of Lepidoptera declined significantly near both of these polluters due to both decreases in species richness and changes in the abundance of individual species, whereas the overall abundance of moths and butterflies was independent of the pollution load. These patterns did not differ between Nikel and Zapolyarnyy, and they were consistent with patterns previously found near the copper–nickel smelter at Monchegorsk. The abundances of Lepidoptera species showed variable changes along pollution gradients, from significantly negative to significantly positive, but individual species showed similar density changes around these three polluters. Disproportional increases in the abundance of a few pollution-tolerant species change the community structure and explain why the overall abundance of moths and butterflies does not decline even in localities experiencing extreme loads of sulphur dioxide and heavy metals.

## 1. Introduction

Studies of pollution impacts on terrestrial biota were originally driven by economic losses in agriculture and forestry rather than by scientific curiosity [1,2]. Consequently, these studies explored acute effects observed near large industrial polluters [3,4,5,6,7,8,9]. To date, the occurrence and importance of acute pollution effects have decreased substantially due to strict emission controls implemented in many countries. As a result, pollution science has shifted from studying local detrimental effects (which still exist in many countries [10,11]) to exploring regional multi-stress effects [12,13]. Nevertheless, the scientific value of studies conducted near big industrial polluters remains high because the landscapes surrounding these polluters offer excellent testing grounds for making observations and inferences about the fundamental ecological processes underlying ecosystem stability in the face of environmental change [14,15]. At the same time, studies conducted in these natural laboratories often lack generality because each polluted area has developed in its own way due to a unique history of events [16,17].

Prior work demonstrated that extreme industrial pollution, which killed forests over hundreds of square kilometres around the copper–nickel smelter at Monchegorsk, in the Murmansk region of Russia, did not change the overall abundance of moths and butterflies (Lepidoptera), although it significantly diminished their diversity [18]. Intriguingly, these findings contrast with the conclusions of a previously published meta-analysis, which did not reveal any significant effect of industrial pollution on arthropod diversity, but instead indicated an increase in the abundance of Lepidoptera in polluted regions [19]. Furthermore, the discovered tolerance of Lepidoptera communities (in terms of overall abundance) to the extreme impacts of industrial pollutants [18] contrasts with alarming reports on the large-scale declines in moths and butterflies because agrochemicals and light pollution, which supposedly drive these declines [20,21,22], do not modify natural ecosystems as dramatically as severe industrial pollution does.

The current study is based on data that had been collected long ago. These data were rescued from file drawers to test the hypothesis that the diversity and overall abundance of moths and butterflies decreases under the impact of industrial pollution in a fragile arctic environment. We also asked (i) whether the strength of the pollution effect on Lepidoptera differs between tundra and forest habitats and (ii) whether individual species show similar density changes around different polluters.

## 2. Materials and Methods

### 2.1. Study Area and Study Sites

**Figure 1:**
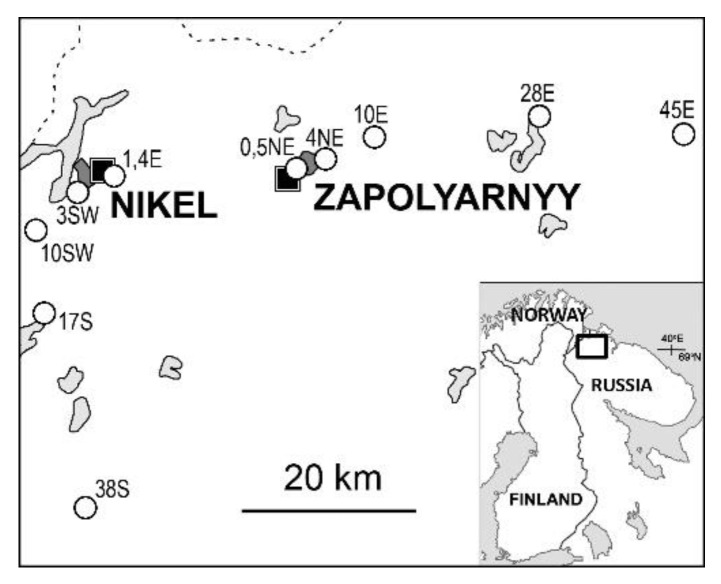
Location of the study sites (circles) in the vicinity of the Nikel copper–nickel smelter and the Zapolyarnyy ore-roasting factory (black squares). The site codes indicate the approximate distance (km) and direction (to the northeast, east, south, or southwest) from the nearest polluter; grey colour indicates lakes. Coordinates of the study sites are provided in Table 2. Insert: The position of the study area in Northern Europe.

Our study area is located in the northwestern part of the Murmansk region in Russia, next to Finland and Norway (Figure 1). In 1920–1944, this territory belonged to Finland. Smelting in Nikel (Kolosjoki at that time) began in 1942. The smelter was partially destroyed by retreating German troops in 1944 and recommenced operations in 1946. The settlement of Zapolyarnyy was established in 1955, in association with the construction of the ore-roasting plant [11]. During the study period (2003–2008), these two polluters (located ca. 25 km apart; Figure 1) emitted into the ambient air similar amounts of sulphur dioxide and of dust containing heavy metals, primarily nickel and copper (Table 1). By contrast, from 1970–1990, the amounts of emitted sulphur dioxide were 4–5-times greater from the smelter at Nikel than from the plant in Zapolyarnyy [11].

The pre-industrial vegetation of the study area [26,27] was dominated by mountain birch (*Betula pubescens* var. *pumila*) woodlands and tundra to the northeast of Zapolyarnyy and by sparse mixed forests of Scots pine (*Pinus sylvestris*) and mountain birch to the south of Nikel. The long-term emissions impact resulted in slight soil acidification and severe contamination by heavy metals [11]. The peak reported metal concentrations in the soils of Nikel and Zapolyarnyy were 3489 and 1020 μg g^−1^, respectively, for copper, and 2990 and 2230 μg g^−1^, respectively, for nickel [10]. The total area affected by air pollution increased from 400 km^2^ in 1973 to more than 3900 km^2^ in 1988, and it has remained this size since that date [28,29]. In the late 1990s, 309 km^2^ around Nikel and 378 km^2^ around Zapolyarnyy were classified as industrial barrens [30].

**Figure 2:**
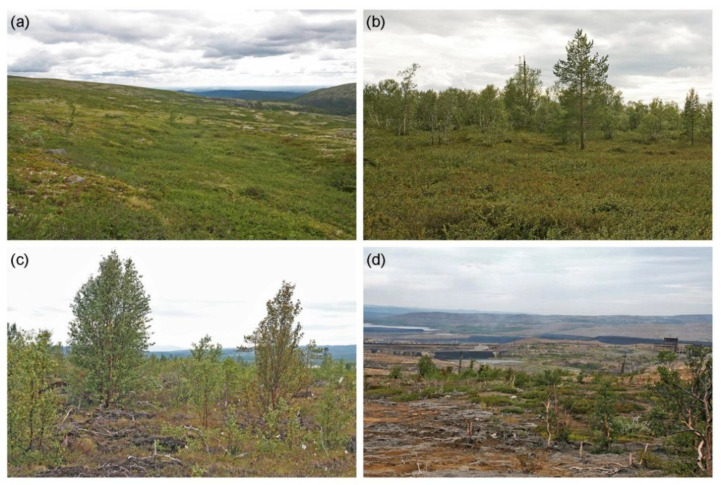
Landscape and the state of vegetation at the study sites: (**a**) site 45 E, unpolluted tundra; (**b**) site 38 S, unpolluted forest; (**c**) site 3 SW, severely damaged forest; (**d**) site 1.4 E, industrial barren. For the positions of the study sites, consult Figure 1 and Table 2.

We systematically selected five sites located 0.5 to 45.3 km from the ore-roasting plant in Zapolyarnyy and five sites located 1.4 to 37.8 km from the copper–nickel smelter at Nikel (Figure 1; Table 2), along the main roads of the study region, to represent different stages of pollution-induced deterioration of natural vegetation (Figure 2). Since the concentrations of pollutants decreased hyperbolically with an increase in the distance from the emission source, we used shorter between-site distances close to the polluters rather than farther away from them (Figure 1). The concentrations of nickel in mountain birch leaves (measured in 2001; Table 2) significantly declined with an increase in the log_10_-transformed distance from the nearest polluter (*r* = −0.90, *n* = 10 sites, *p* = 0.0003), thereby justifying the use of the log_10_-transformed distance as a proxy for pollution load.

The five sites associated with the Zapolyarnyy pollution gradient were selected in tundra containing small patches of mountain birch (Figure 2a), whereas the sites associated with the Nikel pollution gradient represented sparse forests formed by Scots pine and mountain birch (Figure 2b). The vegetation in the most distant sites showed no visible signs of pollution damage (Figure 2a,b). The sites located 3–4 km from the nearest polluter demonstrated stunted growth of woody plants, necrotic damage of birch leaves and Scots pine needles, greatly reduced cover of field-layer vegetation, and early stages of soil erosion (Figure 2c; Table 2). Two sites located next to our polluters were classified as industrial barrens, characterised by sparse patches of vegetation surrounded by bare, eroded soil (Figure 2d). Nevertheless, none of the host plants of our herbivorous Lepidoptera species disappeared completely near these polluters [11].

### 2.2. Collection and Processing

Moths and butterflies were collected once a year in 2003–2005, 2007 (only at sites associated with Nikel), and 2008. During most years, all sites were visited during the same day (between 29 June and 30 July, i.e., during the peak of the flying period), when weather conditions (recorded in the middle of each sampling session) were favourable for moths and butterflies: the ambient air temperature was at least 10 °C; the wind did not exceed three on the Beaufort scale (gentle breeze, 3.4–5.5 m s^−1^); the last rain had fallen at least 2 h before sampling (Data S1).

The protocol of this study differs in some detail from that used in Monchegorsk [18]. Two or three experienced collectors simultaneously searched for adult moths and butterflies and captured them with an entomological net (30 cm in diameter) within a plot 200 × 200 m in size for 30 min, aiming to collect as many of the naturally flying individuals as possible. When no flying moths and butterflies were seen, the collector disturbed them from both low-stature vegetation (herbs and dwarf shrubs) and from branches of trees and shrubs. The collected insects were transported to the laboratory, where M.V.K. identified and counted the individuals of common species and pinned all individuals of rare species and species of uncertain identities. These pinned specimens were identified by †J. Jalava (samples from 2003) and by J. Kullberg (all other samples) and were then deposited in the Zoological Museum, University of Helsinki (MZH). The nomenclature of the species follows [31].

### 2.3. Data Analysis

The abundance of moths and butterflies was quantified by the number of individuals collected from one site during one sampling session; diversity was measured using the Shannon H index. We also estimated the number of species expected in a random sample of 100 individuals for each study site (PAST program [32]).

We explored the spatial variation in the abundance (square-root transformed to normalise the distribution of the residuals) and the diversity of the Lepidoptera in individual samples using linear mixed models (SAS GLIMMIX procedure [33]) with a Gaussian error distribution. In these models, the log_10_-transformed distance from the polluter (covariate), the polluter (Nikel or Zapolyarnyy), and the collector were considered fixed effects, whereas the study site and the collection year were treated as random effects. We adjusted the standard errors and denominator degrees of freedom following [34], and we evaluated the significance of a random effect by testing the likelihood ratio against the *χ*^2^ distribution [35]. The differences in the sample characteristics between collectors were explored using a *t*-test embedded in the GLIMMIX procedure.

The species-level spatial patterns were explored for the 20 most-abundant species. We calculated the effect sizes (ES) by *z*-transforming the Spearman rank correlation coefficients between species abundance and the distance to the nearest polluter. We used meta-analysis [36] to identify the overall pattern and to evaluate similarities between the effects of pollution on the abundance of the same species of moths and butterflies in the Nikel/Zapolyarnyy region (data from the current study) and in the Monchegorsk region (data from [18]).

## 3. Results

### 3.1. Data Overview

We obtained 100 samples of moths and butterflies from 10 study sites (Data S1). These samples contained 2312 individuals of 122 species (Data S2), among which the leafrollers *Epinotia tetraquetrana* (821 individuals), *Ancylis myrtillana* (275 individuals), and *Hedya atropunctana* (259 individuals) were the most abundant. Among-site variations at the time of sampling and in the weather conditions in the middle of a sampling session were not associated with the distance from the nearest polluter (time: *r* = −0.06, *n* = 10 sites, *p* = 0.87; ambient air temperature: *r* = 0.31, *n* = 10 sites, *p* = 0.38; wind speed: *r* = 0.20, *n* = 10 sites, *p* = 0.58; cloudiness: *r* = −0.34, *n* = 10 sites, *p* = 0.33).

### 3.2. Spatial Patterns in Abundance and Diversity

**Figure 3:**
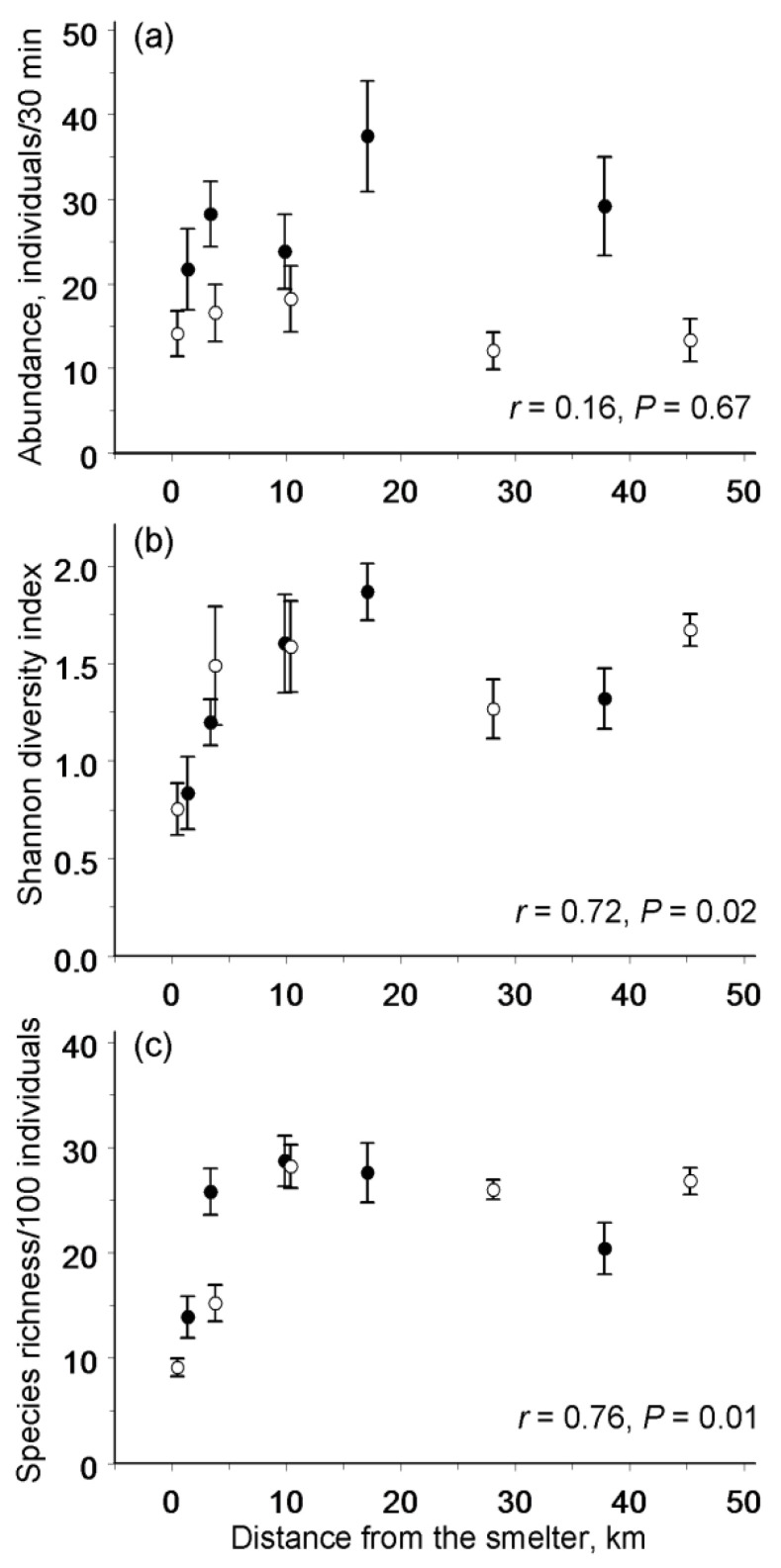
Abundance (**a**), diversity (**b**), and species richness (**c**) of moths and butterflies in relation to the distance from the Nikel copper–nickel smelter (black circles) and from the Zapolyarnyy ore-roasting plant (empty circles). Values are the means (± SE) (**a**,**b**) and the estimated means (± SE) from rarefaction analysis (**c**). Correlations refer to log_10_-transformed distance; the correlation with abundance is based on the square-root-transformed values.

The samples collected in the Nikel (forest) gradient and in the Zapolyarnyy (tundra) gradient did not differ in the abundance of moths and butterflies (Figure 3a; Table 3), in the Shannon diversity index (Figure 3b; Table 3), and in the number of species expected in a random sample of 100 individuals (Figure 3c; *F*_1, 8_ = 0.23, *p* = 0.65). Both the abundance and diversity of Lepidoptera varied significantly among the study sites (random effect in Table 3). However, this variation was explained by the distance to the nearest polluter only for the Shannon diversity index (Figure 3b; Table 3) and for the number of species expected in a sample of 100 individuals (Figure 3c). By contrast, the abundance of moths and butterflies did not change along the pollution gradient (Figure 3a; Table 3). These spatial patterns were consistent between the two polluters (non-significant interaction terms in Table 3). When data from the two most-polluted (barren) sites were excluded, the correlation of both diversity and species richness with the distance from the nearest polluter became non-significant (*r* = 0.17, *n* = 8 sites, *p* = 0.68 and *r* = 0.25, *n* = 8 sites, *p* = 0.55, respectively).

### 3.3. Abundances of Individual Species

**Figure 4:**
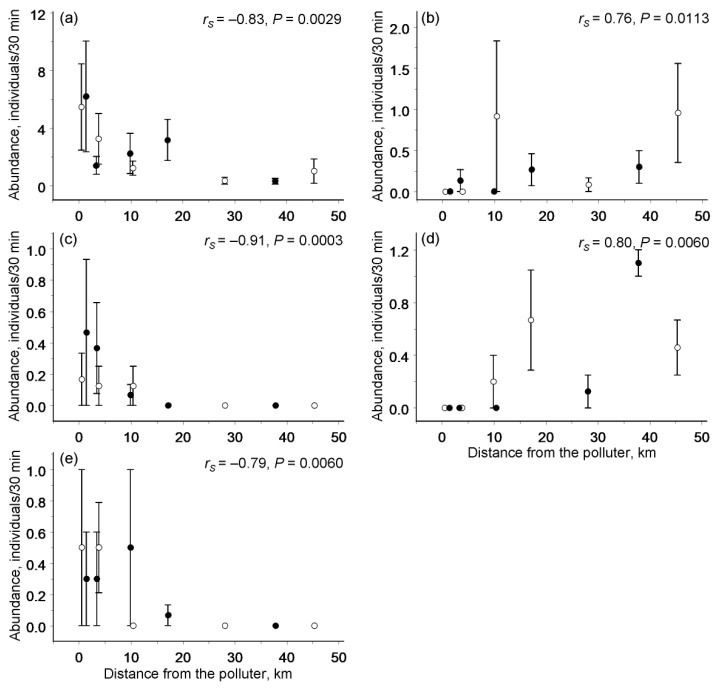
Significant changes in the abundance of individual species of moths and butterflies in relation to the distance from the Nikel copper–nickel smelter (filled circles) and from the Zapolyarnyy ore-roasting plant (empty circles): (**a**) *Hedya atropunctana* (Tortricidae), (**b**) *Agriades optilete* (Lycaenidae), (**c**) *Gesneria centuriella* (Crambidae), (**d**) *Phiaris schulziana* (Tortricidae), and (**e**) *Plutella xylostella* (Plutellidae). Values are the means (± SE) based on year-specific values.

**Figure 5:**
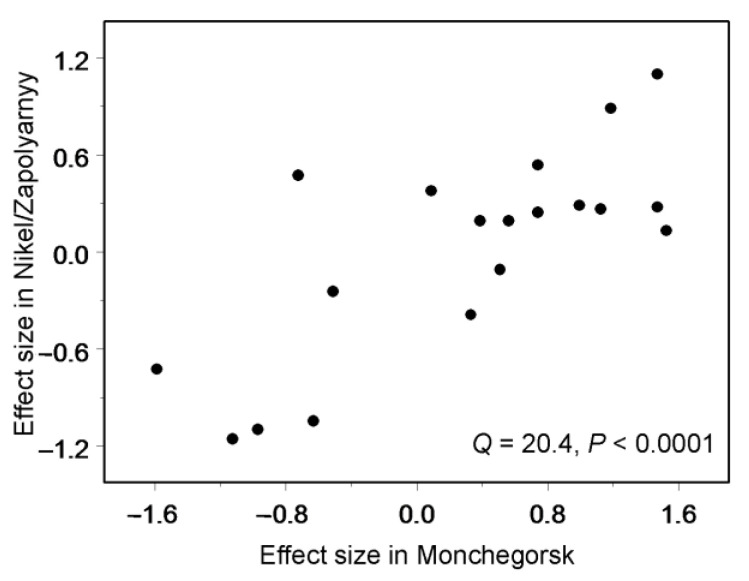
Meta-regression of the effects of distance from the nearest polluter on the abundance of individual Lepidoptera species around Nikel/Zapolyarnyy to the effects on the abundances of the same species around Monchegorsk.

The abundances of 5 of the 20 most frequent species were significantly correlated with the distance from the nearest polluter. The abundances of three species (*H. atropunctana*, *Gesneria centuriella*, and *Plutella xylostella*) increased, whereas the abundances of two species (*Agriades optilete* and *Phiaris schulziana*) declined with increasing proximity to the polluter (Figure 4). By contrast, the abundances of the 15 remaining species were independent of the pollution load. Consistently, meta-analysis demonstrated that, on average, the abundance of individual Lepidoptera species did not correlate with the distance from the nearest polluter (ES = 0.04, *n* = 20 species, CI_95_ = −0.26…0.33), thus matching the community-wide pattern. The effects of distance from the nearest polluter on the abundance of individual Lepidoptera species in the Nikel/Zapolyarnyy region were consistent with the effects observed in the Monchegorsk region (Figure 5).

### 3.4. Research Methodology

The collector’s identity affected neither the characteristics of the individual samples (main effect in Table 3) nor the spatial patterns invoked from these samples (collector × distance interaction in Table 3). The samples, which were simultaneously collected by V.Z. and M.V.K., contained similar numbers of individuals (21.9 and 23.2, respectively; *t* = 1.53, *p* = 0.13) and yielded similar values for the Shannon diversity index (1.38 and 1.43, respectively; *t* = 0.95, *p* = 0. 35). Furthermore, the characteristics of the individual samples were significantly correlated with each other (abundance: *r* = 0.93, *n* = 45 sampling sessions, *p* < 0.0001; diversity: *r* = 0.84, *n* = 45 sampling sessions, *p* < 0.0001).

## 4. Discussion

### 4.1. Spatial Patterns in Abundance and Diversity

In agreement with our expectations, the diversity and species richness of the Lepidoptera communities declined significantly near the copper–nickel smelter at Nikel and near the ore-roasting plant in Zapolyarnyy. By contrast, the overall abundance of moths and butterflies was independent of the environmental contamination near both polluters. These patterns did not differ between Nikel and Zapolyarnyy, even though these polluters are located in different vegetation zones (forest and tundra, respectively), and they are consistent with patterns previously [18] found near the copper-nickel smelter at Monchegorsk. The discovered consistency in the changes in the Lepidoptera communities among the three pollution gradients (at Monchegorsk, Nikel, and Zapolyarnyy) suggests that the patterns observed at Monchegorsk [18] were not restricted to that particular polluted area. However, an earlier meta-analysis [19] did not detect any pollution effect on the diversity of terrestrial arthropods, but revealed the significant increase in the abundance of moths and butterflies (most of which were free-living herbivores) near industrial polluters. Therefore, the question to answer is why the patterns found in our studies differ from the general patterns detected by the cited meta-analysis.

The significant increase in the abundance of moths and butterflies near industrial polluters has been previously attributed to research bias, i.e., to the tendency to collect data on organisms or under conditions in which one has an expectation of detecting significant effects. This bias likely emerged from the preferential documentation of the density changes of forest pest species favoured by industrial polluters, because these species impose additional threats to forests [19]. By contrast, our findings on the absence of a pollution impact on the abundance of moths and butterflies near three industrial polluters are based on community-wide data, which include all observed species irrespective of their response to pollution.

The overall abundance of moths and butterflies remains stable along pollution gradients because the abundances of several Lepidoptera species increase sharply near the polluters (in line with the pattern discovered by meta-analysis [19]), while the populations of other species decline (Figure 4). The among-species variation discovered in the responses to pollution (and to associated environmental stressors) is likely a common trait of insects, because both winners and losers have been previously identified by analyses of climate-driven changes in communities of dragonflies [37], moths [38], and bumblebees [39]. Importantly, ours is the first study to demonstrate that the responses of individual species to industrial pollution were consistent between the two polluted regions, Nikel/Zapolyarnyy (this study) and Monchegorsk [18], despite the substantial differences in their pre-industrial environments and pollution histories. This finding indirectly supports our earlier conclusion [18] that the responses of individual Lepidoptera species to pollution could be predicted based on their particular traits. In particular, the abundances of monophagous species that fed inside live plant tissues and hibernated as imagoes or pupae were not affected by pollution from the Monchegorsk smelter, whereas the abundances of oligophagous and polyphagous species that fed externally on plants and hibernated as larvae generally declined near that smelter [18].

The decrease in diversity observed in Lepidoptera in both the Monchegorsk and Nikel/Zapolyarnyy regions was repeatedly suggested to represent a general ecosystem response to environmental stress [40,41]. However, meta-analyses revealed that the diversity near industrial polluters decreased in fungi, bryophytes, and vascular plants, but not in terrestrial arthropods [42]. The explanation for the discrepancy between the results of the meta-analysis [19] and our current findings is likely rooted in the extremely high level of habitat deterioration near Monchegorsk, Nikel, and Zapolyarnyy. In these regions, the diversity of Lepidoptera decreases only in extremely polluted habitats (termed industrial barrens), where the soil concentrations of nickel and copper range from 1000–9000 μg g^−1^ [10]. Industrial barrens have been reported to occur only near 36 polluters worldwide [10], so the vast majority of arthropod diversity studies included in the meta-analysis were conducted in pollution gradients lacking industrial barrens. Therefore, the outcomes of the meta-analysis [19] should not be compared with the entire Monchegorsk and Nikel/Zapolyarnyy pollution gradients, but only with the outer parts of these gradients (excluding industrial barrens), where the diversity of moths and butterflies did not change with the pollution load ([18] and the present study). Thus, the consistent decrease in the diversity of moths and butterflies near Monchegorsk, Nikel, and Zapolyarnyy should be viewed as a response to the extreme levels of industrial pollution, and this response results from both decreases in species richness and changes in the relative abundances of the persisting species in favour of a few pollution-tolerant species.

### 4.2. Research Methodology

The previously published study addressing the impacts of aerial emissions from the Monchegorsk copper–nickel smelter on Lepidoptera communities [18] was based on data collected during 1162 person-hours, i.e., the data collection required nearly ten months of highly skilled work, taking into account the time spent for the transportation of the collectors to/from the study sites. These great investments of time and resources make repetition of this work unlikely. Consequently, the question arises as to whether a less extensive data collection programme could yield reliable conclusions regarding the responses of Lepidoptera communities to environmental pollution.

Shortening the sampling session from two to one person-hour and the concomitant work of two collectors allowed a great decrease in the time investments in the field work. The current study is based on data collected during 50 person-hours. Nevertheless, these data appeared sufficient to reveal the same spatial patterns in abundance and diversity of moths and butterflies along the pollution gradients as were previously discovered (based on much greater efforts) in the Monchegorsk region [18]. Furthermore, we found that the collector’s identity did not affect the spatial patterns observed in the communities of moths and butterflies. Therefore, we conclude that the netting of insects during a fixed period of time is well suited for monitoring Lepidoptera communities along environmental gradients.

Manzano and Julier [43] recently argued that the original specimens, rather than their recorded identifications, constitute the primary data of ecological and evolutionary studies reporting species-level data. Our method involves the collection of all individuals of Lepidoptera and, thus, potentially allows depositing of these individuals in relevant repositories. In this respect, our collection method for butterflies and larger moths has a better agreement with FAIR principles (which advocate that scientific data should be findable, accessible, interoperable, and reusable [44]) than was observed with transect counting [45], which is widely used in monitoring programmes all around the world [46,47], despite its generation of non-controllable data. Nevertheless, we preserved only a few vouchers of common, easily identifiable species.

## 5. Conclusions

The discovery of an astonishingly low reproducibility of published scientific studies [48] forces reconsideration of the importance of replicated studies. From this perspective, our study of a new dataset, which yielded the same conclusions as a previously published study [18], may have even greater value than the original innovative study. The main importance of the current study lies in the finding that data from two polluted regions (located some 190 km apart) demonstrated the same changes in the diversity and abundance of Lepidoptera in response to an increase in pollution load. These changes included (1) an absence of pollution effects on overall abundance, (2) a significant decrease in diversity limited to the most-polluted (barren) sites, and (3) concordance between the two polluted regions in terms of the strength of pollution effects on the abundances of individual species.

## Figures and Tables

**Table 1:** Atmospheric emissions of principal pollutants from the copper–nickel smelter at Nikel and from the ore-roasting plant in Zapolyarnyy (metric tons) from 2003 to 2008 (after [11,23,24,25]). Data on nickel and copper emissions for 2006–2008 are not available.

Year	Nikel			Zapolyarnyy		
	SO_2_	Ni	Cu	SO_2_	Ni	Cu
2003	60,600	149	83	63,700	180	84
2004	56,400	154	86	56,000	175	82
2005	55,500	157	88	51,400	171	84
2006	57,600	.	.	50,800	.	.
2007	62,700	.	.	44,600	.	.
2008	53,800	.	.	45,800	.	.

**Table 2:** Characteristics of study sites (after [11]).

Polluter	Site ^a^	Latitude, N	Longitude, E	Distance from the Polluter, km	Ni in Birch Leaves, μg g^−1^	Stand Basal Area, m^2^ ha^−1^	Cover of Field Layer Plants, %
Nikel	1.4 E	69°25′	30°17′	1.4	273	0.7	1.1
	3 SW	69°24′	30°11′	3.4	97	1.0	7.4
	10 SW	69°21′	30°03′	9.9	48	4.3	19.7
	17 S	69°16′	30°05′	17.1	29	2.0	49.0
	38 S	69°04′	30°12′	37.8	33	11.7	25.3
Zapolyarnyy	0.5 NE	69°25′	30°48′	0.5	366	0	0
	4 NE	69°26′	30°53′	3.8	68	0.3	2.5
	10 E	69°27′	31°02′	10.4	36	2.0	45.2
	28 E	69°28′	31°30′	28.1	15	0.7	33.0
	45 E	69°25′	31°57′	45.3	8	0	39.0

^a^ The site codes indicate approximate distance from the nearest polluter in km and the direction to the northeast, east, south, or southwest of the polluter.

**Table 3:** Sources of variation in the abundance and diversity of Lepidoptera (SAS GLIMMIX procedure, type 3 tests).

Effect Type	Explanatory Variable	Diversity		Abundance	
		Test Statistics	*p*	Test Statistics	*p*
Fixed	Polluter (P)	*F*_1, 7.50_ = 0.21	0.66	*F*_1, 9.34_ = 1.05	0.33
	Collector (C)	*F*_2, 78.3_ = 0.15	0.86	*F*_2, 78.4_ = 1.39	0.26
	Distance (D)	*F*_1, 7.48_ = 5.30	0.05	*F*_1, 9.31_ = 1.16	0.31
	P × C	*F*_2, 78.1_ = 1.23	0.380	*F*_2, 78.3_ = 0.06	0.95
	C × D	*F*_2, 78.1_ = 0.16	0.85	*F*_2, 78.3_ = 0.39	0.68
	P × D	*F*_1, 7.49_ = 0.08	0.79	*F*_1, 9.33_ = 2.42	0.15
	P × C × D	*F*_2, 78.1_ = 1.13	0.33	*F*_2, 78.3_ = 0.33	0.72
Random	Site	*χ*^2^_1_ = 13.6	0.0001	*χ*^2^_1_ = 3.63	0.03
	Year	*χ*^2^_1_ = 59.3	<0.0001	*χ*^2^_1_ = 92.6	<0.0001

## Data Availability

All data are included in this publication as the Supplementary Material.

## Supplementary Materials

The following Supporting Information can be downloaded at: https://www.mdpi.com/article/10.3390/insects13121124/s1, Data S1: Sample characteristics; Data S2: Abundances of individual species in each sample.
