# Supplementary material for: Diversity but Not Overall Abundance of Moths and Butterflies (Insecta: Lepidoptera) Decreases around Two Arctic Polluters
Source: Insects. 2022 Dec 5;13(12):1124. doi: 10.3390/insects13121124 (PMC9786165; doi:10.3390/insects13121124)
Supplement: Supplementary file 1 [file insects-13-01124-s001.zip › insects-2011804-supplementary.pdf]

Diversity but not overall abundance of moths and butterflies (Insecta: Lepidoptera) decreases around two arctic polluters

Mikhail V. Kozlov*, Vitali Zverev and Elena L. Zvereva

[1]*Department of Biology, University of Turku, FI-20014 Turku, Finland*

*Corresponding author. E-mail address: mikoz@utu.fi

Supplementary data

Data S1
Sample characteristics.

<u>Meta-data</u>

Column 1: Collection year.
Column 2: Collection site (consult Fig. 2 and Table S2).
Column 3: Day of collection (1 = 1 January).
Column 4: Hour of collection.
Column 5: Ambient air temperature, °C.
Column 6: Wind speed (Beaufort scale).
Column 7: Cloudiness (%).
Column 8: Collector's code.
Column 9: Number of collected individuals.
Column 10: Shannon H diversity index.

```
2003 0.5ne 180 20 16 2 0 mk 28 0.822
2003 0.5ne 180 20 16 2 0 vz 19 0.708
2003 1.4e 181 12 20 0 0 mk 30 0.531
2003 1.4e 181 12 20 0 0 vz 22 1.245
2003 10e 180 16 17 1 0 mk 18 2.077
2003 10e 180 16 17 1 0 vz 8 1.733
2003 10sw 180 20 18 0 0 mk 38 2.111
2003 10sw 180 20 18 0 0 vz 42 1.821
2003 17s 181 20 22 0 0 mk 35 1.971
2003 17s 181 20 22 0 0 vz 35 2.119
2003 28e 180 16 16 1 0 mk 14 1.128
2003 28e 180 16 16 1 0 vz 15 1.679
2003 38s 181 18 22 0 0 mk 36 1.035
2003 38s 181 18 22 0 0 vz 38 1.249
2003 3sw 180 18 17 1 0 mk 24 2.395
2003 3sw 180 18 17 1 0 vz 19 2.260
2003 45e 180 14 14 1 0 mk 6 1.561
2003 45e 180 14 14 1 0 vz 9 1.581
2003 4ne 180 22 17 2 0 mk 28 1.738
2003 4ne 180 22 17 2 0 vz 33 1.703
2004 0.5ne 188 18 20 2 50 em 19 1.297
2004 0.5ne 188 18 20 2 50 mk 11 1.288
2004 0.5ne 188 18 20 2 50 vz 7 0.683
2004 1.4e 189 22 20 1 0 em 24 1.368
2004 1.4e 189 22 20 1 0 mk 23 1.409
2004 1.4e 189 22 20 1 0 vz 20 1.708
2004 10e 188 16 20 2 0 em 22 2.011
2004 10e 188 16 20 2 0 mk 29 2.000
2004 10e 188 16 20 2 0 vz 28 2.104
2004 10sw 188 22 18 1 50 em 41 2.352
2004 10sw 188 22 18 1 50 mk 29 1.768
2004 10sw 188 22 18 1 50 vz 35 2.077
```

2004 17s 189 20 20 1 0 em 59 2.021
2004 17s 189 20 20 1 0 mk 53 2.316
2004 17s 189 20 20 1 0 vz 52 2.059
2004 28e 188 16 20 3 0 em 18 1.706
2004 28e 188 16 20 3 0 mk 11 1.666
2004 28e 188 16 20 3 0 vz 6 1.792
2004 38s 189 18 20 1 50 em 42 1.507
2004 38s 189 18 20 1 50 mk 30 1.598
2004 38s 189 18 20 1 50 vz 30 1.847
2004 3sw 188 20 20 3 0 em 30 1.355
2004 3sw 188 20 20 3 0 mk 16 1.700
2004 3sw 188 20 20 3 0 vz 21 1.566
2004 45e 188 14 20 3 0 em 16 1.527
2004 45e 188 14 20 3 0 mk 15 2.154
2004 45e 188 14 20 3 0 vz 9 1.523
2004 4ne 188 20 20 2 75 em 45 1.048
2004 4ne 188 20 20 2 75 mk 49 1.465
2004 4ne 188 20 20 2 75 vz 34 1.324
2005 0.5ne 182 14 13 1 75 mk 21 0.945
2005 0.5ne 182 14 13 1 75 vz 18 1.038
2005 1.4e 181 18 12 1 100 mk 52 0.696
2005 1.4e 181 20 12 1 100 vz 52 0.594
2005 10e 182 16 15 1 75 mk 45 2.019
2005 10e 182 16 15 1 75 vz 27 2.339
2005 10sw 181 20 12 1 100 mk 18 1.904
2005 10sw 181 20 12 1 100 vz 25 1.355
2005 17s 180 20 15 1 100 mk 64 1.520
2005 17s 180 20 15 1 100 vz 67 1.318
2005 28e 182 18 15 2 0 mk 28 1.615
2005 28e 182 18 15 2 0 vz 28 1.329
2005 38s 180 18 14 1 50 mk 55 1.008
2005 38s 180 18 14 1 50 vz 61 1.112
2005 3sw 181 20 13 1 100 mk 31 1.964
2005 3sw 181 22 13 1 100 vz 32 1.833
2005 45e 182 18 13 2 75 mk 29 1.897
2005 45e 182 18 13 2 75 vz 28 2.279
2005 4ne 182 16 13 1 100 mk 40 1.583
2005 4ne 182 16 13 1 100 vz 23 1.249
2007 1.4e 185 22 20 0 0 mk 10 0.898
2007 1.4e 185 22 20 0 0 vz 12 1.352
2007 10sw 185 20 20 1 1 mk 14 1.909
2007 10sw 185 20 20 1 1 vz 17 2.313
2007 17s 185 18 20 0 1 mk 23 2.364
2007 17s 185 18 20 0 1 vz 15 2.303
2007 38s 185 18 20 0 0 mk 17 2.150
2007 38s 185 18 20 0 0 vz 10 1.887
2007 3sw 185 20 20 1 0 mk 23 2.219
2007 3sw 185 20 20 1 0 vz 15 1.894
2008 0.5ne 211 10 9 0 50 mk 6 0.868
2008 0.5ne 211 10 9 0 50 vz 3 0.000
2008 1.4e 211 10 9 0 50 mk 1 0.000
2008 1.4e 211 10 9 0 50 vz 1 0.000
2008 10e 211 12 10 1 100 mk 3 1.099
2008 10e 211 12 10 1 100 vz 1 0.000
2008 10sw 210 20 12 1 0 mk 1 0.000
2008 10sw 210 20 12 1 0 vz 2 0.000
2008 17s 210 10 12 2 0 mk 9 1.889
2008 17s 210 10 12 2 0 vz 6 1.011
2008 28e 211 12 11 1 100 mk 3 0.636
2008 28e 211 12 11 1 100 vz 4 1.040
2008 38s 210 18 13 2 0 mk 3 0.636
2008 38s 210 18 13 2 0 vz 2 0.693
2008 3sw 210 20 11 2 50 mk 1 0.000
2008 3sw 210 20 11 2 50 vz 0 0.000
2008 45e 211 14 12 1 50 mk 6 1.330
2008 45e 211 14 12 1 50 vz 9 1.677
2008 4ne 211 12 11 1 75 mk 16 0.602
2008 4ne 211 12 11 1 75 vz 3 0.636

Data S2
Abundances of individual species in each sample.

<u>Meta-data</u>

Column 1: Collection year.
Column 2: Collection site (consult Fig. 2 and Table S2).
Column 3: Day of collection (1 = 1 January).
Column 4: Hour of collection.
Column 5: Ambient air temperature, °C.
Column 6: Wind speed (Beaufort scale).
Column 7: Cloudiness (0, 0%; 1, 25%; 2, 50%; 3, 75%; 4, 100%).
Column 8: Collector's code.
Column 9: Species name.
Column 10: Number of collected individuals.

```
2003 45e 180 14 14 1 0 mk Glyphipterix_haworthana_(Stephens,_1834) 1
2003 45e 180 14 14 1 0 mk Ancylis_myrtillana_(Treitschke,_1830) 2
2003 45e 180 14 14 1 0 mk Rheumaptera_subhastata_(Nolcken,_1870) 1
2003 45e 180 14 14 1 0 mk Pammene_clanculana_(Tengström,_1869) 1
2003 45e 180 14 14 1 0 mk Coranarta_cordigera_(Thunberg,_1788) 1
2003 45e 180 14 14 1 0 vz Erebia_pandrose_(Borkhausen,_1788) 1
2003 45e 180 14 14 1 0 vz Rheumaptera_subhastata_(Nolcken,_1870) 1
2003 45e 180 14 14 1 0 vz Ancylis_myrtillana_(Treitschke,_1830) 4
2003 45e 180 14 14 1 0 vz Ancylis_unguicella_(Linnaeus,_1758) 1
2003 45e 180 14 14 1 0 vz Hedya_atropunctana_(Zetterstedt,_1839) 1
2003 45e 180 14 14 1 0 vz Callisto_coffeella_(Zetterstedt,_1839) 1
2003 28e 180 16 16 1 0 mk Ancylis_myrtillana_(Treitschke,_1830) 9
2003 28e 180 16 16 1 0 mk Phiaris_schulziana_(Fabricius,_1776) 1
2003 28e 180 16 16 1 0 mk Pammene_clanculana_(Tengström,_1869) 1
2003 28e 180 16 16 1 0 mk Apotomis_sauciana_(Frölich,_1828) 2
2003 28e 180 16 16 1 0 mk Aethes_deutschiana_(Zetterstedt,_1839) 1
2003 28e 180 16 16 1 0 vz Erebia_pandrose_(Borkhausen,_1788) 2
2003 28e 180 16 16 1 0 vz Ancylis_myrtillana_(Treitschke,_1830) 6
2003 28e 180 16 16 1 0 vz Xanthorhoe_annotinata_(Zetterstedt,_1839) 1
2003 28e 180 16 16 1 0 vz Macaria_carbonaria_(Clerck,_1759) 1
2003 28e 180 16 16 1 0 vz Eulia_ministrana_(Linnaeus,_1758) 1
2003 28e 180 16 16 1 0 vz Chionodes_viduella_(Fabricius,_1794) 1
2003 28e 180 16 16 1 0 vz Pammene_clanculana_(Tengström,_1869) 3
2003 10e 180 16 17 1 0 mk Micropterix_aureatella_(Scopoli,_1763) 5
2003 10e 180 16 17 1 0 mk Hedya_atropunctana_(Zetterstedt,_1839) 1
2003 10e 180 16 17 1 0 mk Ancylis_myrtillana_(Treitschke,_1830) 3
2003 10e 180 16 17 1 0 mk Ancylis_unguicella_(Linnaeus,_1758) 1
2003 10e 180 16 17 1 0 mk Melitaea_athalia_(Rottemburg,_1775) 1
2003 10e 180 16 17 1 0 mk Eupithecia_satyrata_(Hübner,_1813) 1
2003 10e 180 16 17 1 0 mk Xanthorhoe_annotinata_(Zetterstedt,_1839) 1
2003 10e 180 16 17 1 0 mk Udea_inquinatalis_(Lienig_&_Zeller,_1846) 1
2003 10e 180 16 17 1 0 mk Adela_cuprella_(Denis_&_Schiffermüller,_1775) 1
2003 10e 180 16 17 1 0 mk Udea_decrepitalis_(Herrich-Schäffer,_1848) 3
2003 10e 180 16 17 1 0 vz Argyroploce_lediana_(Linnaeus,_1758) 1
2003 10e 180 16 17 1 0 vz Ancylis_myrtillana_(Treitschke,_1830) 2
2003 10e 180 16 17 1 0 vz Hedya_atropunctana_(Zetterstedt,_1839) 1
2003 10e 180 16 17 1 0 vz Denisia_obscurella_(Brandt,_1937) 1
2003 10e 180 16 17 1 0 vz Spargania_luctuata_(Denis_&_Schiffermüller,_1775) 1
2003 10e 180 16 17 1 0 vz Udea_inquinatalis_(Lienig_&_Zeller,_1846) 2
2003 4ne 180 22 17 2 0 mk Epinotia_tetraquetrana_(Haworth,_1811) 10
2003 4ne 180 22 17 2 0 mk Ancylis_unguicella_(Linnaeus,_1758) 2
2003 4ne 180 22 17 2 0 mk Apotomis_fraterculana_Krogerus,_1946 1
2003 4ne 180 22 17 2 0 mk Gypsonoma_nitidulana_(Lienig_&_Zeller,_1846) 1
2003 4ne 180 22 17 2 0 mk Dahlica_lazuri_(Clerck,_1759) 1
2003 4ne 180 22 17 2 0 mk Paraswammerdamia_conspersella_(Tengström,_1848) 7
2003 4ne 180 22 17 2 0 mk Ancylis_myrtillana_(Treitschke,_1830) 3
2003 4ne 180 22 17 2 0 mk Hedya_atropunctana_(Zetterstedt,_1839) 3
2003 4ne 180 22 17 2 0 vz Epinotia_tetraquetrana_(Haworth,_1811) 12
2003 4ne 180 22 17 2 0 vz Ancylis_myrtillana_(Treitschke,_1830) 3
```

2003 4ne 180 22 17 2 0 vz Hedya_atropunctana_(Zetterstedt,_1839) 8
2003 4ne 180 22 17 2 0 vz Paraswammerdamia_conspersella_(Tengström,_1848) 5
2003 4ne 180 22 17 2 0 vz Parornix_loganella_(Stainton,_1848) 1
2003 4ne 180 22 17 2 0 vz Apotomis_moestana_(Wocke,_1862) 1
2003 4ne 180 22 17 2 0 vz Prolita_sexpunctella_(Fabricius,_1794) 1
2003 4ne 180 22 17 2 0 vz Ancylis_uncella_(Denis_&_Schiffermüller,_1775) 2
2003 0.5ne 180 20 16 2 0 mk Hedya_atropunctana_(Zetterstedt,_1839) 14
2003 0.5ne 180 20 16 2 0 mk Epinotia_tetraquetrana_(Haworth,_1811) 13
2003 0.5ne 180 20 16 2 0 mk Swammerdamia_passerella_(Zetterstedt,_1839) 1
2003 0.5ne 180 20 16 2 0 vz Hedya_atropunctana_(Zetterstedt,_1839) 14
2003 0.5ne 180 20 16 2 0 vz Epinotia_tetraquetrana_(Haworth,_1811) 4
2003 0.5ne 180 20 16 2 0 vz Ancylis_myrtillana_(Treitschke,_1830) 1
2003 1.4e 181 12 20 0 0 mk Rheumaptera_hastata_(Linnaeus,_1758) 1
2003 1.4e 181 12 20 0 0 mk Hedya_atropunctana_(Zetterstedt,_1839) 26
2003 1.4e 181 12 20 0 0 mk Ancylis_myrtillana_(Treitschke,_1830) 1
2003 1.4e 181 12 20 0 0 mk Epinotia_tetraquetrana_(Haworth,_1811) 2
2003 1.4e 181 12 20 0 0 vz Hedya_atropunctana_(Zetterstedt,_1839) 15
2003 1.4e 181 12 20 0 0 vz Ancylis_myrtillana_(Treitschke,_1830) 1
2003 1.4e 181 12 20 0 0 vz Glyphipterix_haworthana_(Stephens,_1834) 1
2003 1.4e 181 12 20 0 0 vz Argyroploce_lediana_(Linnaeus,_1758) 1
2003 1.4e 181 12 20 0 0 vz Epinotia_tetraquetrana_(Haworth,_1811) 1
2003 1.4e 181 12 20 0 0 vz Coleophora_virgaureae_Stainton,_1857 1
2003 1.4e 181 12 20 0 0 vz Pseudotelphusa_paripunctella_(Thunberg,_1794) 1
2003 1.4e 181 12 20 0 0 vz Swammerdamia_caesiella_(Hübner,_1796) 1
2003 3sw 180 18 17 1 0 mk Udea_inquinatalis_(Lienig_&_Zeller,_1846) 3
2003 3sw 180 18 17 1 0 mk Hedya_atropunctana_(Zetterstedt,_1839) 3
2003 3sw 180 18 17 1 0 mk Argyroploce_lediana_(Linnaeus,_1758) 5
2003 3sw 180 18 17 1 0 mk Elophos_vittaria_(Thunberg,_1788) 2
2003 3sw 180 18 17 1 0 mk Ancylis_myrtillana_(Treitschke,_1830) 1
2003 3sw 180 18 17 1 0 mk Aethes_deutschiana_(Zetterstedt,_1839) 2
2003 3sw 180 18 17 1 0 mk Epinotia_tetraquetrana_(Haworth,_1811) 2
2003 3sw 180 18 17 1 0 mk Phiaris_palustrana_(Lienig_&_Zeller,_1846) 1
2003 3sw 180 18 17 1 0 mk Epinotia_crenana_(Hübner,_1799) 1
2003 3sw 180 18 17 1 0 mk Ancylis_unguicella_(Linnaeus,_1758) 1
2003 3sw 180 18 17 1 0 mk Phiaris_obsoletana_(Zetterstedt,_1839) 1
2003 3sw 180 18 17 1 0 mk Choristoneura_albaniana_(Walker,_1863) 1
2003 3sw 180 18 17 1 0 mk Syndemis_musculana_(Hübner,_1799) 1
2003 3sw 180 18 17 1 0 vz Udea_inquinatalis_(Lienig_&_Zeller,_1846) 2
2003 3sw 180 18 17 1 0 vz Glyphipterix_haworthana_(Stephens,_1834) 1
2003 3sw 180 18 17 1 0 vz Stictea_mygindiana_(Denis_&_Schiffermüller,_1775) 1
2003 3sw 180 18 17 1 0 vz Hedya_atropunctana_(Zetterstedt,_1839) 4
2003 3sw 180 18 17 1 0 vz Syndemis_musculana_(Hübner,_1799) 1
2003 3sw 180 18 17 1 0 vz Ancylis_myrtillana_(Treitschke,_1830) 2
2003 3sw 180 18 17 1 0 vz Argyroploce_lediana_(Linnaeus,_1758) 1
2003 3sw 180 18 17 1 0 vz Argyroploce_concretana_(Wocke,_1862) 1
2003 3sw 180 18 17 1 0 vz Gesneria_centuriella_(Denis_&_Schiffermüller,_1775) 1
2003 3sw 180 18 17 1 0 vz Choristoneura_albaniana_(Walker,_1863) 3
2003 3sw 180 18 17 1 0 vz Spargania_luctuata_(Denis_&_Schiffermüller,_1775) 2
2003 10sw 180 20 18 0 0 mk Micropterix_aureatella_(Scopoli,_1763) 14
2003 10sw 180 20 18 0 0 mk Boloria_euphrosyne_(Linnaeus,_1758) 5
2003 10sw 180 20 18 0 0 mk Ancylis_myrtillana_(Treitschke,_1830) 4
2003 10sw 180 20 18 0 0 mk Elophos_vittaria_(Thunberg,_1788) 1
2003 10sw 180 20 18 0 0 mk Glyphipterix_haworthana_(Stephens,_1834) 1
2003 10sw 180 20 18 0 0 mk Choristoneura_albaniana_(Walker,_1863) 2
2003 10sw 180 20 18 0 0 mk Argyroploce_lediana_(Linnaeus,_1758) 2
2003 10sw 180 20 18 0 0 mk Neofaculta_infernella_(Herrich-Schäffer,_1854) 1
2003 10sw 180 20 18 0 0 mk Scopula_ternata_(Schrank,_1802) 1
2003 10sw 180 20 18 0 0 mk Ancylis_unguicella_(Linnaeus,_1758) 1
2003 10sw 180 20 18 0 0 mk Ematurga_atomaria_(Linnaeus,_1758) 3
2003 10sw 180 20 18 0 0 mk Aphelia_viburnana_(Denis_&_Schiffermüller,_1775) 2
2003 10sw 180 20 18 0 0 mk Pammene_clanculana_(Tengström,_1869) 1
2003 10sw 180 20 18 0 0 vz Boloria_euphrosyne_(Linnaeus,_1758) 14
2003 10sw 180 20 18 0 0 vz Xanthorhoe_annotinata_(Zetterstedt,_1839) 1
2003 10sw 180 20 18 0 0 vz Ematurga_atomaria_(Linnaeus,_1758) 1
2003 10sw 180 20 18 0 0 vz Aphelia_viburnana_(Denis_&_Schiffermüller,_1775) 1
2003 10sw 180 20 18 0 0 vz Micropterix_aureatella_(Scopoli,_1763) 4
2003 10sw 180 20 18 0 0 vz Hedya_atropunctana_(Zetterstedt,_1839) 13
2003 10sw 180 20 18 0 0 vz Argyroploce_lediana_(Linnaeus,_1758) 2
2003 10sw 180 20 18 0 0 vz Eriopsela_quadrana_(Hübner,_1813) 1
2003 10sw 180 20 18 0 0 vz Ancylis_myrtillana_(Treitschke,_1830) 3
2003 10sw 180 20 18 0 0 vz Neofaculta_infernella_(Herrich-Schäffer,_1854) 1

2003 10sw 180 20 18 0 0 vz Choristoneura_albaniana_(Walker,_1863) 1
2003 17s 181 20 22 0 0 mk Rheumaptera_subhastata_(Nolcken,_1870) 2
2003 17s 181 20 22 0 0 mk Boloria_freija_(Thunberg,_1791) 1
2003 17s 181 20 22 0 0 mk Ematurga_atomaria_(Linnaeus,_1758) 1
2003 17s 181 20 22 0 0 mk Xanthorhoe_spadicearia_(Denis_&_Schiffermüller,_1775) 2
2003 17s 181 20 22 0 0 mk Eulia_ministrana_(Linnaeus,_1758) 1
2003 17s 181 20 22 0 0 mk Hedya_atropunctana_(Zetterstedt,_1839) 2
2003 17s 181 20 22 0 0 mk Micropterix_aureatella_(Scopoli,_1763) 2
2003 17s 181 20 22 0 0 mk Udea_inquinatalis_(Lienig_&_Zeller,_1846) 2
2003 17s 181 20 22 0 0 mk Epinotia_tetraquetrana_(Haworth,_1811) 2
2003 17s 181 20 22 0 0 mk Ancylis_myrtillana_(Treitschke,_1830) 16
2003 17s 181 20 22 0 0 mk Gypsonoma_nitidulana_(Lienig_&_Zeller,_1846) 2
2003 17s 181 20 22 0 0 mk Choristoneura_albaniana_(Walker,_1863) 2
2003 17s 181 20 22 0 0 vz Choristoneura_albaniana_(Walker,_1863) 6
2003 17s 181 20 22 0 0 vz Ancylis_myrtillana_(Treitschke,_1830) 6
2003 17s 181 20 22 0 0 vz Aethes_deutschiana_(Zetterstedt,_1839) 1
2003 17s 181 20 22 0 0 vz Hedya_atropunctana_(Zetterstedt,_1839) 8
2003 17s 181 20 22 0 0 vz Euphydryas_iduna_(Dalman,_1816) 1
2003 17s 181 20 22 0 0 vz Epinotia_tetraquetrana_(Haworth,_1811) 6
2003 17s 181 20 22 0 0 vz Falcaria_lacertinaria_(Linnaeus,_1758) 1
2003 17s 181 20 22 0 0 vz Micropterix_aureatella_(Scopoli,_1763) 2
2003 17s 181 20 22 0 0 vz Eupithecia_satyrata_(Hübner,_1813) 1
2003 17s 181 20 22 0 0 vz Eupithecia_intricata_(Zetterstedt,_1839) 1
2003 17s 181 20 22 0 0 vz Aphelia_viburnana_(Denis_&_Schiffermüller,_1775) 1
2003 17s 181 20 22 0 0 vz Udea_inquinatalis_(Lienig_&_Zeller,_1846) 1
2003 38s 181 18 22 0 0 mk Epinotia_tetraquetrana_(Haworth,_1811) 27
2003 38s 181 18 22 0 0 mk Ancylis_myrtillana_(Treitschke,_1830) 1
2003 38s 181 18 22 0 0 mk Phiaris_schulziana_(Fabricius,_1776) 1
2003 38s 181 18 22 0 0 mk Pammene_clanculana_(Tengström,_1869) 1
2003 38s 181 18 22 0 0 mk Xanthorhoe_annotinata_(Zetterstedt,_1839) 2
2003 38s 181 18 22 0 0 mk Aphelia_viburnana_(Denis_&_Schiffermüller,_1775) 2
2003 38s 181 18 22 0 0 mk Ancylis_diminutana_(Haworth,_1811) 1
2003 38s 181 18 22 0 0 mk Taleporia_tubulosa_(Retzius,_1783) 1
2003 38s 181 18 22 0 0 vz Macaria_fusca_(Thunberg,_1792) 1
2003 38s 181 18 22 0 0 vz Choristoneura_albaniana_(Walker,_1863) 2
2003 38s 181 18 22 0 0 vz Boloria_euphrosyne_(Linnaeus,_1758) 3
2003 38s 181 18 22 0 0 vz Boloria_freija_(Thunberg,_1791) 1
2003 38s 181 18 22 0 0 vz Rheumaptera_hastata_(Linnaeus,_1758) 1
2003 38s 181 18 22 0 0 vz Stigmella_salicis_(Stainton,_1854) 1
2003 38s 181 18 22 0 0 vz Epinotia_tetraquetrana_(Haworth,_1811) 26
2003 38s 181 18 22 0 0 vz Ancylis_myrtillana_(Treitschke,_1830) 2
2003 38s 181 18 22 0 0 vz Phiaris_schulziana_(Fabricius,_1776) 1
2004 45e 188 14 20 3 0 mk Zygaena_exulans_(Hohenwarth,_1792) 2
2004 45e 188 14 20 3 0 mk Agriades_optilete_(Knoch,_1781) 2
2004 45e 188 14 20 3 0 mk Aphelia_viburnana_(Denis_&_Schiffermüller,_1775) 1
2004 45e 188 14 20 3 0 mk Ancylis_myrtillana_(Treitschke,_1830) 4
2004 45e 188 14 20 3 0 mk Phiaris_schulziana_(Fabricius,_1776) 1
2004 45e 188 14 20 3 0 mk Chionodes_viduella_(Fabricius,_1794) 1
2004 45e 188 14 20 3 0 mk Pammene_clanculana_(Tengström,_1869) 1
2004 45e 188 14 20 3 0 mk Prolita_sexpunctella_(Fabricius,_1794) 1
2004 45e 188 14 20 3 0 mk Phiaris_turfosana_(Herrich-Schäffer,_1851) 1
2004 45e 188 14 20 3 0 mk Sparganothis_rubicundana_(Herrich-Schäffer,_1856) 1
2004 45e 188 14 20 3 0 vz Zygaena_exulans_(Hohenwarth,_1792) 3
2004 45e 188 14 20 3 0 vz Agriades_optilete_(Knoch,_1781) 2
2004 45e 188 14 20 3 0 vz Ancylis_myrtillana_(Treitschke,_1830) 2
2004 45e 188 14 20 3 0 vz Boloria_freija_(Thunberg,_1791) 1
2004 45e 188 14 20 3 0 vz Udea_decrepitalis_(Herrich-Schäffer,_1848) 1
2004 45e 188 14 20 3 0 em Zygaena_exulans_(Hohenwarth,_1792) 1
2004 45e 188 14 20 3 0 em Aphelia_viburnana_(Denis_&_Schiffermüller,_1775) 1
2004 45e 188 14 20 3 0 em Ancylis_myrtillana_(Treitschke,_1830) 8
2004 45e 188 14 20 3 0 em Pammene_clanculana_(Tengström,_1869) 3
2004 45e 188 14 20 3 0 em Sympistis_heliophila_(Paykull,_1793) 1
2004 45e 188 14 20 3 0 em Phiaris_septentrionana_(Curtis,_1835) 1
2004 45e 188 14 20 3 0 em Glacies_coracina_(Esper,_1805) 1
2004 28e 188 16 20 3 0 mk Rheumaptera_subhastata_(Nolcken,_1870) 1
2004 28e 188 16 20 3 0 mk Phiaris_obsoletana_(Zetterstedt,_1839) 1
2004 28e 188 16 20 3 0 mk Ancylis_myrtillana_(Treitschke,_1830) 5
2004 28e 188 16 20 3 0 mk Hedya_atropunctana_(Zetterstedt,_1839) 1
2004 28e 188 16 20 3 0 mk Aphelia_viburnana_(Denis_&_Schiffermüller,_1775) 1
2004 28e 188 16 20 3 0 mk Glacies_coracina_(Esper,_1805) 1
2004 28e 188 16 20 3 0 mk Paraswammerdamia_conspersella_(Tengström,_1848) 1

2004 28e 188 16 20 3 0 vz Phiaris_septentrionana_(Curtis,_1835) 1
2004 28e 188 16 20 3 0 vz Ancylis_myrtillana_(Treitschke,_1830) 1
2004 28e 188 16 20 3 0 vz Erebia_pandrose_(Borkhausen,_1788) 1
2004 28e 188 16 20 3 0 vz Boloria_frigga_(Thunberg,_1791) 1
2004 28e 188 16 20 3 0 vz Chionodes_viduella_(Fabricius,_1794) 1
2004 28e 188 16 20 3 0 vz Aethes_deutschiana_(Zetterstedt,_1839) 1
2004 28e 188 16 20 3 0 em Phiaris_obsoletana_(Zetterstedt,_1839) 1
2004 28e 188 16 20 3 0 em Ancylis_myrtillana_(Treitschke,_1830) 8
2004 28e 188 16 20 3 0 em Agriades_optilete_(Knoch,_1781) 1
2004 28e 188 16 20 3 0 em Zygaena_exulans_(Hohenwarth,_1792) 1
2004 28e 188 16 20 3 0 em Phiaris_bipunctana_(Fabricius,_1794) 2
2004 28e 188 16 20 3 0 em Eupithecia_satyrata_(Hübner,_1813) 1
2004 28e 188 16 20 3 0 em Pammene_clanculana_(Tengström,_1869) 3
2004 28e 188 16 20 3 0 em Syngrapha_parilis_(Hübner,_1809) 1
2004 10e 188 16 20 2 0 mk Boloria_euphrosyne_(Linnaeus,_1758) 1
2004 10e 188 16 20 2 0 mk Agriades_optilete_(Knoch,_1781) 2
2004 10e 188 16 20 2 0 mk Argyroploce_lediana_(Linnaeus,_1758) 4
2004 10e 188 16 20 2 0 mk Epinotia_tetraquetrana_(Haworth,_1811) 8
2004 10e 188 16 20 2 0 mk Ancylis_myrtillana_(Treitschke,_1830) 1
2004 10e 188 16 20 2 0 mk Scopula_ternata_(Schrank,_1802) 5
2004 10e 188 16 20 2 0 mk Eulia_ministrana_(Linnaeus,_1758) 1
2004 10e 188 16 20 2 0 mk Hedya_atropunctana_(Zetterstedt,_1839) 5
2004 10e 188 16 20 2 0 mk Spargania_luctuata_(Denis_&_Schiffermüller,_1775) 1
2004 10e 188 16 20 2 0 mk Pammene_clanculana_(Tengström,_1869) 1
2004 10e 188 16 20 2 0 vz Boloria_euphrosyne_(Linnaeus,_1758) 1
2004 10e 188 16 20 2 0 vz Boloria_aquilonaris_(Stichel,_1908) 2
2004 10e 188 16 20 2 0 vz Agriades_optilete_(Knoch,_1781) 8
2004 10e 188 16 20 2 0 vz Zygaena_exulans_(Hohenwarth,_1792) 1
2004 10e 188 16 20 2 0 vz Argyroploce_lediana_(Linnaeus,_1758) 4
2004 10e 188 16 20 2 0 vz Epinotia_tetraquetrana_(Haworth,_1811) 2
2004 10e 188 16 20 2 0 vz Ancylis_myrtillana_(Treitschke,_1830) 1
2004 10e 188 16 20 2 0 vz Scopula_ternata_(Schrank,_1802) 5
2004 10e 188 16 20 2 0 vz Xanthorhoe_abrasaria_(Herrich-Schäffer,_1855) 2
2004 10e 188 16 20 2 0 vz Boloria_eunomia_(Esper,_1800) 1
2004 10e 188 16 20 2 0 vz Phiaris_turfosana_(Herrich-Schäffer,_1851) 1
2004 10e 188 16 20 2 0 em Ancylis_unguicella_(Linnaeus,_1758) 1
2004 10e 188 16 20 2 0 em Boloria_euphrosyne_(Linnaeus,_1758) 2
2004 10e 188 16 20 2 0 em Agriades_optilete_(Knoch,_1781) 1
2004 10e 188 16 20 2 0 em Argyroploce_lediana_(Linnaeus,_1758) 1
2004 10e 188 16 20 2 0 em Epinotia_tetraquetrana_(Haworth,_1811) 6
2004 10e 188 16 20 2 0 em Scopula_ternata_(Schrank,_1802) 4
2004 10e 188 16 20 2 0 em Eulia_ministrana_(Linnaeus,_1758) 3
2004 10e 188 16 20 2 0 em Hedya_atropunctana_(Zetterstedt,_1839) 2
2004 10e 188 16 20 2 0 em Celypha_lacunana_(Denis_&_Schiffermüller,_1775) 2
2004 4ne 188 20 20 2 75 mk Falcaria_lacertinaria_(Linnaeus,_1758) 1
2004 4ne 188 20 20 2 75 mk Aethes_smeathmanniana_(Fabricius,_1781) 1
2004 4ne 188 20 20 2 75 mk Hedya_atropunctana_(Zetterstedt,_1839) 6
2004 4ne 188 20 20 2 75 mk Argyroploce_lediana_(Linnaeus,_1758) 1
2004 4ne 188 20 20 2 75 mk Ancylis_myrtillana_(Treitschke,_1830) 7
2004 4ne 188 20 20 2 75 mk Epinotia_tetraquetrana_(Haworth,_1811) 27
2004 4ne 188 20 20 2 75 mk Pammene_clanculana_(Tengström,_1869) 4
2004 4ne 188 20 20 2 75 mk Prolita_sexpunctella_(Fabricius,_1794) 1
2004 4ne 188 20 20 2 75 mk Ancylis_unguicella_(Linnaeus,_1758) 1
2004 4ne 188 20 20 2 75 vz Hedya_atropunctana_(Zetterstedt,_1839) 9
2004 4ne 188 20 20 2 75 vz Ancylis_myrtillana_(Treitschke,_1830) 2
2004 4ne 188 20 20 2 75 vz Epinotia_tetraquetrana_(Haworth,_1811) 17
2004 4ne 188 20 20 2 75 vz Scopula_ternata_(Schrank,_1802) 1
2004 4ne 188 20 20 2 75 vz Pammene_clanculana_(Tengström,_1869) 4
2004 4ne 188 20 20 2 75 vz Prolita_sexpunctella_(Fabricius,_1794) 1
2004 4ne 188 20 20 2 75 em Hedya_atropunctana_(Zetterstedt,_1839) 6
2004 4ne 188 20 20 2 75 em Ancylis_myrtillana_(Treitschke,_1830) 4
2004 4ne 188 20 20 2 75 em Epinotia_tetraquetrana_(Haworth,_1811) 31
2004 4ne 188 20 20 2 75 em Plutella_xylostella_(Linnaeus,_1758) 2
2004 4ne 188 20 20 2 75 em Pammene_clanculana_(Tengström,_1869) 1
2004 4ne 188 20 20 2 75 em Ancylis_unguicella_(Linnaeus,_1758) 1
2004 0.5ne 188 18 20 2 50 mk Hedya_atropunctana_(Zetterstedt,_1839) 2
2004 0.5ne 188 18 20 2 50 mk Epinotia_tetraquetrana_(Haworth,_1811) 5
2004 0.5ne 188 18 20 2 50 mk Plutella_xylostella_(Linnaeus,_1758) 2
2004 0.5ne 188 18 20 2 50 mk Gesneria_centuriella_(Denis_&_Schiffermüller,_1775) 2
2004 0.5ne 188 18 20 2 50 vz Hedya_atropunctana_(Zetterstedt,_1839) 4
2004 0.5ne 188 18 20 2 50 vz Epinotia_tetraquetrana_(Haworth,_1811) 3

2004 0.5ne 188 18 20 2 50 em Hedya_atropunctana_(Zetterstedt,_1839) 7
2004 0.5ne 188 18 20 2 50 em Epinotia_tetraquetrana_(Haworth,_1811) 6
2004 0.5ne 188 18 20 2 50 em Plutella_xylostella_(Linnaeus,_1758) 4
2004 0.5ne 188 18 20 2 50 em Ancylis_myrtillana_(Treitschke,_1830) 2
2004 1.4e 189 22 20 1 0 mk Gesneria_centuriella_(Denis_&_Schiffermüller,_1775) 3
2004 1.4e 189 22 20 1 0 mk Hedya_atropunctana_(Zetterstedt,_1839) 8
2004 1.4e 189 22 20 1 0 mk Epinotia_tetraquetrana_(Haworth,_1811) 9
2004 1.4e 189 22 20 1 0 mk Ancylis_myrtillana_(Treitschke,_1830) 1
2004 1.4e 189 22 20 1 0 mk Eupithecia_satyrata_(Hübner,_1813) 1
2004 1.4e 189 22 20 1 0 mk Ancylis_unguicella_(Linnaeus,_1758) 1
2004 1.4e 189 22 20 1 0 vz Gesneria_centuriella_(Denis_&_Schiffermüller,_1775) 3
2004 1.4e 189 22 20 1 0 vz Mompha_idaei_(Zeller,_1839) 3
2004 1.4e 189 22 20 1 0 vz Hedya_atropunctana_(Zetterstedt,_1839) 4
2004 1.4e 189 22 20 1 0 vz Epinotia_tetraquetrana_(Haworth,_1811) 7
2004 1.4e 189 22 20 1 0 vz Ancylis_myrtillana_(Treitschke,_1830) 1
2004 1.4e 189 22 20 1 0 vz Pammene_clanculana_(Tengström,_1869) 1
2004 1.4e 189 22 20 1 0 vz Ancylis_unguicella_(Linnaeus,_1758) 1
2004 1.4e 189 22 20 1 0 em Gesneria_centuriella_(Denis_&_Schiffermüller,_1775) 1
2004 1.4e 189 22 20 1 0 em Hedya_atropunctana_(Zetterstedt,_1839) 12
2004 1.4e 189 22 20 1 0 em Epinotia_tetraquetrana_(Haworth,_1811) 7
2004 1.4e 189 22 20 1 0 em Ancylis_myrtillana_(Treitschke,_1830) 1
2004 1.4e 189 22 20 1 0 em Eupithecia_gelidata_Möschler,_1860 1
2004 1.4e 189 22 20 1 0 em Pammene_clanculana_(Tengström,_1869) 1
2004 1.4e 189 22 20 1 0 em Apotomis_sororculana_(Zetterstedt,_1839) 1
2004 3sw 188 20 20 3 0 mk Scopula_ternata_(Schrank,_1802) 2
2004 3sw 188 20 20 3 0 mk Ancylis_myrtillana_(Treitschke,_1830) 3
2004 3sw 188 20 20 3 0 mk Epinotia_tetraquetrana_(Haworth,_1811) 4
2004 3sw 188 20 20 3 0 mk Gesneria_centuriella_(Denis_&_Schiffermüller,_1775) 1
2004 3sw 188 20 20 3 0 mk Hedya_atropunctana_(Zetterstedt,_1839) 4
2004 3sw 188 20 20 3 0 mk Argyroploce_lediana_(Linnaeus,_1758) 2
2004 3sw 188 20 20 3 0 vz Ancylis_myrtillana_(Treitschke,_1830) 3
2004 3sw 188 20 20 3 0 vz Aphelia_viburnana_(Denis_&_Schiffermüller,_1775) 1
2004 3sw 188 20 20 3 0 vz Epinotia_tetraquetrana_(Haworth,_1811) 11
2004 3sw 188 20 20 3 0 vz Hedya_atropunctana_(Zetterstedt,_1839) 2
2004 3sw 188 20 20 3 0 vz Boloria_euphrosyne_(Linnaeus,_1758) 1
2004 3sw 188 20 20 3 0 vz Eupithecia_intricata_(Zetterstedt,_1839) 1
2004 3sw 188 20 20 3 0 vz Prolita_sexpunctella_(Fabricius,_1794) 1
2004 3sw 188 20 20 3 0 vz Ancylis_unguicella_(Linnaeus,_1758) 1
2004 3sw 188 20 20 3 0 em Agriades_optilete_(Knoch,_1781) 2
2004 3sw 188 20 20 3 0 em Scopula_ternata_(Schrank,_1802) 3
2004 3sw 188 20 20 3 0 em Ancylis_myrtillana_(Treitschke,_1830) 3
2004 3sw 188 20 20 3 0 em Aphelia_viburnana_(Denis_&_Schiffermüller,_1775) 2
2004 3sw 188 20 20 3 0 em Epinotia_tetraquetrana_(Haworth,_1811) 18
2004 3sw 188 20 20 3 0 em Perizoma_albulata_(Denis_&_Schiffermüller,_1775) 1
2004 3sw 188 20 20 3 0 em Prolita_sexpunctella_(Fabricius,_1794) 1
2004 10sw 188 22 18 1 50 mk Stictea_mygindiana_(Denis_&_Schiffermüller,_1775) 1
2004 10sw 188 22 18 1 50 mk Scopula_ternata_(Schrank,_1802) 2
2004 10sw 188 22 18 1 50 mk Sympistis_heliophila_(Paykull,_1793) 1
2004 10sw 188 22 18 1 50 mk Grapholita_aureolana_Tengström,_1848 1
2004 10sw 188 22 18 1 50 mk Epinotia_tetraquetrana_(Haworth,_1811) 5
2004 10sw 188 22 18 1 50 mk Hedya_atropunctana_(Zetterstedt,_1839) 1
2004 10sw 188 22 18 1 50 mk Ancylis_myrtillana_(Treitschke,_1830) 14
2004 10sw 188 22 18 1 50 mk Pleurota_bicostella_(Clerck,_1759) 1
2004 10sw 188 22 18 1 50 mk Xanthorhoe_annotinata_(Zetterstedt,_1839) 1
2004 10sw 188 22 18 1 50 mk Pammene_clanculana_(Tengström,_1869) 1
2004 10sw 188 22 18 1 50 mk Ancylis_comptana_(Frölich,_1828) 1
2004 10sw 188 22 18 1 50 vz Scopula_ternata_(Schrank,_1802) 5
2004 10sw 188 22 18 1 50 vz Xanthorhoe_abrasaria_(Herrich-Schäffer,_1855) 2
2004 10sw 188 22 18 1 50 vz Grapholita_aureolana_Tengström,_1848 1
2004 10sw 188 22 18 1 50 vz Falcaria_lacertinaria_(Linnaeus,_1758) 1
2004 10sw 188 22 18 1 50 vz Epinotia_tetraquetrana_(Haworth,_1811) 4
2004 10sw 188 22 18 1 50 vz Argyroploce_lediana_(Linnaeus,_1758) 1
2004 10sw 188 22 18 1 50 vz Hedya_atropunctana_(Zetterstedt,_1839) 4
2004 10sw 188 22 18 1 50 vz Phiaris_bipunctana_(Fabricius,_1794) 1
2004 10sw 188 22 18 1 50 vz Ancylis_myrtillana_(Treitschke,_1830) 12
2004 10sw 188 22 18 1 50 vz Eupithecia_satyrata_(Hübner,_1813) 1
2004 10sw 188 22 18 1 50 vz Cydia_cognatana_(Barrett,_1874) 1
2004 10sw 188 22 18 1 50 vz Pammene_clanculana_(Tengström,_1869) 2
2004 10sw 188 22 18 1 50 em Stictea_mygindiana_(Denis_&_Schiffermüller,_1775) 5
2004 10sw 188 22 18 1 50 em Scopula_ternata_(Schrank,_1802) 2
2004 10sw 188 22 18 1 50 em Xanthorhoe_abrasaria_(Herrich-Schäffer,_1855) 2

2004 10sw 188 22 18 1 50 em Grapholita_aureolana_Tengström,_1848 1
2004 10sw 188 22 18 1 50 em Ancylis_unguicella_(Linnaeus,_1758) 4
2004 10sw 188 22 18 1 50 em Epinotia_tetraquetrana_(Haworth,_1811) 3
2004 10sw 188 22 18 1 50 em Syndemis_musculana_(Hübner,_1799) 1
2004 10sw 188 22 18 1 50 em Argyroploce_lediana_(Linnaeus,_1758) 2
2004 10sw 188 22 18 1 50 em Coleophora_glitzella_O._Hofmann,_1869 1
2004 10sw 188 22 18 1 50 em Hedya_atropunctana_(Zetterstedt,_1839) 9
2004 10sw 188 22 18 1 50 em Ancylis_myrtillana_(Treitschke,_1830) 7
2004 10sw 188 22 18 1 50 em Spargania_luctuata_(Denis_&_Schiffermüller,_1775) 1
2004 10sw 188 22 18 1 50 em Gesneria_centuriella_(Denis_&_Schiffermüller,_1775) 1
2004 10sw 188 22 18 1 50 em Pammene_clanculana_(Tengström,_1869) 2
2004 17s 189 20 20 1 0 mk Pammene_clanculana_(Tengström,_1869) 1
2004 17s 189 20 20 1 0 mk Phiaris_palustrana_(Lienig_&_Zeller,_1846) 1
2004 17s 189 20 20 1 0 mk Coleophora_glitzella_O._Hofmann,_1869 3
2004 17s 189 20 20 1 0 mk Eupithecia_intricata_(Zetterstedt,_1839) 2
2004 17s 189 20 20 1 0 mk Scopula_ternata_(Schrank,_1802) 4
2004 17s 189 20 20 1 0 mk Eupithecia_satyrata_(Hübner,_1813) 1
2004 17s 189 20 20 1 0 mk Hedya_atropunctana_(Zetterstedt,_1839) 3
2004 17s 189 20 20 1 0 mk Eupithecia_gelidata_Möschler,_1860 1
2004 17s 189 20 20 1 0 mk Ancylis_myrtillana_(Treitschke,_1830) 21
2004 17s 189 20 20 1 0 mk Epinotia_tetraquetrana_(Haworth,_1811) 3
2004 17s 189 20 20 1 0 mk Chionodes_continuella_(Zeller,_1839) 1
2004 17s 189 20 20 1 0 mk Xanthorhoe_abrasaria_(Herrich-Schäffer,_1855) 2
2004 17s 189 20 20 1 0 mk Eulia_ministrana_(Linnaeus,_1758) 1
2004 17s 189 20 20 1 0 mk Neofaculta_infernella_(Herrich-Schäffer,_1854) 4
2004 17s 189 20 20 1 0 mk Phiaris_schulziana_(Fabricius,_1776) 1
2004 17s 189 20 20 1 0 mk Phiaris_bipunctana_(Fabricius,_1794) 1
2004 17s 189 20 20 1 0 mk Plutella_xylostella_(Linnaeus,_1758) 1
2004 17s 189 20 20 1 0 mk Ancylis_unguicella_(Linnaeus,_1758) 1
2004 17s 189 20 20 1 0 mk Coleophora_vacciniella_Herrich-Schäffer,_1861 1
2004 17s 189 20 20 1 0 vz Pammene_clanculana_(Tengström,_1869) 1
2004 17s 189 20 20 1 0 vz Argyroploce_concretana_(Wocke,_1862) 1
2004 17s 189 20 20 1 0 vz Sparganothis_rubicundana_(Herrich-Schäffer,_1856) 1
2004 17s 189 20 20 1 0 vz Agriades_optilete_(Knoch,_1781) 1
2004 17s 189 20 20 1 0 vz Eupithecia_intricata_(Zetterstedt,_1839) 1
2004 17s 189 20 20 1 0 vz Scopula_ternata_(Schrank,_1802) 1
2004 17s 189 20 20 1 0 vz Hedya_atropunctana_(Zetterstedt,_1839) 10
2004 17s 189 20 20 1 0 vz Ancylis_myrtillana_(Treitschke,_1830) 15
2004 17s 189 20 20 1 0 vz Udea_inquinatalis_(Lienig_&_Zeller,_1846) 1
2004 17s 189 20 20 1 0 vz Epinotia_tetraquetrana_(Haworth,_1811) 13
2004 17s 189 20 20 1 0 vz Neofaculta_infernella_(Herrich-Schäffer,_1854) 2
2004 17s 189 20 20 1 0 vz Syndemis_musculana_(Hübner,_1799) 1
2004 17s 189 20 20 1 0 vz Ancylis_unguicella_(Linnaeus,_1758) 1
2004 17s 189 20 20 1 0 vz Celypha_lacunana_(Denis_&_Schiffermüller,_1775) 1
2004 17s 189 20 20 1 0 vz Xanthorhoe_montanata_(Denis_&_Schiffermüller,_1775) 1
2004 17s 189 20 20 1 0 vz Xanthorhoe_spadicearia_(Denis_&_Schiffermüller,_1775) 1
2004 17s 189 20 20 1 0 em Pammene_clanculana_(Tengström,_1869) 1
2004 17s 189 20 20 1 0 em Coleophora_glitzella_O._Hofmann,_1869 2
2004 17s 189 20 20 1 0 em Scopula_ternata_(Schrank,_1802) 3
2004 17s 189 20 20 1 0 em Hedya_atropunctana_(Zetterstedt,_1839) 9
2004 17s 189 20 20 1 0 em Ancylis_myrtillana_(Treitschke,_1830) 25
2004 17s 189 20 20 1 0 em Epinotia_tetraquetrana_(Haworth,_1811) 7
2004 17s 189 20 20 1 0 em Xanthorhoe_abrasaria_(Herrich-Schäffer,_1855) 1
2004 17s 189 20 20 1 0 em Filatima_incomptella_(Herrich-Schäffer,_1854) 1
2004 17s 189 20 20 1 0 em Neofaculta_infernella_(Herrich-Schäffer,_1854) 2
2004 17s 189 20 20 1 0 em Stictea_mygindiana_(Denis_&_Schiffermüller,_1775) 2
2004 17s 189 20 20 1 0 em Ancylis_uncella_(Denis_&_Schiffermüller,_1775) 1
2004 17s 189 20 20 1 0 em Syndemis_musculana_(Hübner,_1799) 1
2004 17s 189 20 20 1 0 em Rheumaptera_subhastata_(Nolcken,_1870) 1
2004 17s 189 20 20 1 0 em Spargania_luctuata_(Denis_&_Schiffermüller,_1775) 1
2004 17s 189 20 20 1 0 em Argyroploce_lediana_(Linnaeus,_1758) 1
2004 17s 189 20 20 1 0 em Boloria_eunomia_(Esper,_1800) 1
2004 38s 189 18 20 1 50 mk Agriades_optilete_(Knoch,_1781) 1
2004 38s 189 18 20 1 50 mk Ancylis_myrtillana_(Treitschke,_1830) 2
2004 38s 189 18 20 1 50 mk Epinotia_tetraquetrana_(Haworth,_1811) 10
2004 38s 189 18 20 1 50 mk Scopula_ternata_(Schrank,_1802) 11
2004 38s 189 18 20 1 50 mk Phiaris_schulziana_(Fabricius,_1776) 1
2004 38s 189 18 20 1 50 mk Hedya_atropunctana_(Zetterstedt,_1839) 1
2004 38s 189 18 20 1 50 mk Clepsis_senecionana_(Hübner,_1819) 1
2004 38s 189 18 20 1 50 mk Phiaris_obsoletana_(Zetterstedt,_1839) 3
2004 38s 189 18 20 1 50 vz Apotomis_sororculana_(Zetterstedt,_1839) 1

2004 38s 189 18 20 1 50 vz Ematurga_atomaria_(Linnaeus,_1758) 1
2004 38s 189 18 20 1 50 vz Boloria_euphrosyne_(Linnaeus,_1758) 3
2004 38s 189 18 20 1 50 vz Agriades_optilete_(Knoch,_1781) 2
2004 38s 189 18 20 1 50 vz Ancylis_myrtillana_(Treitschke,_1830) 1
2004 38s 189 18 20 1 50 vz Epinotia_tetraquetrana_(Haworth,_1811) 12
2004 38s 189 18 20 1 50 vz Scopula_ternata_(Schrank,_1802) 6
2004 38s 189 18 20 1 50 vz Boloria_freija_(Thunberg,_1791) 1
2004 38s 189 18 20 1 50 vz Sympistis_heliophila_(Paykull,_1793) 1
2004 38s 189 18 20 1 50 vz Pammene_clanculana_(Tengström,_1869) 2
2004 38s 189 18 20 1 50 em Phiaris_schulziana_(Fabricius,_1776) 2
2004 38s 189 18 20 1 50 em Hedya_atropunctana_(Zetterstedt,_1839) 2
2004 38s 189 18 20 1 50 em Micropterix_aureatella_(Scopoli,_1763) 1
2004 38s 189 18 20 1 50 em Eupithecia_intricata_(Zetterstedt,_1839) 1
2004 38s 189 18 20 1 50 em Sympistis_heliophila_(Paykull,_1793) 1
2004 38s 189 18 20 1 50 em Coleophora_vacciniella_Herrich-Schäffer,_1861 1
2004 38s 189 18 20 1 50 em Ancylis_myrtillana_(Treitschke,_1830) 5
2004 38s 189 18 20 1 50 em Epinotia_tetraquetrana_(Haworth,_1811) 23
2004 38s 189 18 20 1 50 em Scopula_ternata_(Schrank,_1802) 6
2005 45e 182 18 13 2 75 MK Glacies_coracina_(Esper,_1805) 6
2005 45e 182 18 13 2 75 MK Choristoneura_albaniana_(Walker,_1863) 1
2005 45e 182 18 13 2 75 MK Phiaris_schulziana_(Fabricius,_1776) 1
2005 45e 182 18 13 2 75 MK Glyphipterix_haworthana_(Stephens,_1834) 5
2005 45e 182 18 13 2 75 MK Hedya_atropunctana_(Zetterstedt,_1839) 3
2005 45e 182 18 13 2 75 MK Ancylis_myrtillana_(Treitschke,_1830) 4
2005 45e 182 18 13 2 75 MK Epinotia_tetraquetrana_(Haworth,_1811) 7
2005 45e 182 18 13 2 75 MK Pammene_clanculana_(Tengström,_1869) 2
2005 45e 182 18 13 2 75 VZ Macaria_carbonaria_(Clerck,_1759) 2
2005 45e 182 18 13 2 75 VZ Glacies_coracina_(Esper,_1805) 4
2005 45e 182 18 13 2 75 VZ Hedya_atropunctana_(Zetterstedt,_1839) 2
2005 45e 182 18 13 2 75 VZ Phiaris_schulziana_(Fabricius,_1776) 1
2005 45e 182 18 13 2 75 VZ Ancylis_myrtillana_(Treitschke,_1830) 1
2005 45e 182 18 13 2 75 VZ Xanthorhoe_annotinata_(Zetterstedt,_1839) 1
2005 45e 182 18 13 2 75 VZ Udea_inquinatalis_(Lienig_&_Zeller,_1846) 1
2005 45e 182 18 13 2 75 VZ Choristoneura_albaniana_(Walker,_1863) 1
2005 45e 182 18 13 2 75 VZ Hedya_atropunctana_(Zetterstedt,_1839) 1
2005 45e 182 18 13 2 75 VZ Phiaris_turfosana_(Herrich-Schäffer,_1851) 1
2005 45e 182 18 13 2 75 VZ Apotomis_sororculana_(Zetterstedt,_1839) 1
2005 45e 182 18 13 2 75 VZ Epinotia_tetraquetrana_(Haworth,_1811) 9
2005 45e 182 18 13 2 75 VZ Pammene_clanculana_(Tengström,_1869) 2
2005 45e 182 18 13 2 75 VZ Glyphipterix_haworthana_(Stephens,_1834) 1
2005 28e 182 18 15 2 0 MK Rheumaptera_subhastata_(Nolcken,_1870) 2
2005 28e 182 18 15 2 0 MK Ancylis_myrtillana_(Treitschke,_1830) 2
2005 28e 182 18 15 2 0 MK Glyphipterix_haworthana_(Stephens,_1834) 1
2005 28e 182 18 15 2 0 MK Epinotia_tetraquetrana_(Haworth,_1811) 15
2005 28e 182 18 15 2 0 MK Hedya_atropunctana_(Zetterstedt,_1839) 2
2005 28e 182 18 15 2 0 MK Apotomis_sauciana_(Frölich,_1828) 3
2005 28e 182 18 15 2 0 MK Apotomis_lemniscatana_(Kennel,_1901) 1
2005 28e 182 18 15 2 0 MK Pammene_clanculana_(Tengström,_1869) 1
2005 28e 182 18 15 2 0 MK Choristoneura_albaniana_(Walker,_1863) 1
2005 28e 182 18 15 2 0 VZ Glacies_coracina_(Esper,_1805) 1
2005 28e 182 18 15 2 0 VZ Macaria_carbonaria_(Clerck,_1759) 1
2005 28e 182 18 15 2 0 VZ Glyphipterix_haworthana_(Stephens,_1834) 1
2005 28e 182 18 15 2 0 VZ Epinotia_tetraquetrana_(Haworth,_1811) 13
2005 28e 182 18 15 2 0 VZ Apotomis_sauciana_(Frölich,_1828) 1
2005 28e 182 18 15 2 0 VZ Xanthorhoe_annotinata_(Zetterstedt,_1839) 1
2005 28e 182 18 15 2 0 VZ Sympistis_heliophila_(Paykull,_1793) 1
2005 28e 182 18 15 2 0 VZ Chionodes_viduella_(Fabricius,_1794) 1
2005 10e 182 16 15 1 75 MK Ancylis_myrtillana_(Treitschke,_1830) 1
2005 10e 182 16 15 1 75 MK Udea_decrepitalis_(Herrich-Schäffer,_1848) 1
2005 10e 182 16 15 1 75 MK Hedya_atropunctana_(Zetterstedt,_1839) 2
2005 10e 182 16 15 1 75 MK Eulia_ministrana_(Linnaeus,_1758) 1
2005 10e 182 16 15 1 75 MK Scopula_ternata_(Schrank,_1802) 2
2005 10e 182 16 15 1 75 MK Eupithecia_satyrata_(Hübner,_1813) 1
2005 10e 182 16 15 1 75 MK Syndemis_musculana_(Hübner,_1799) 1
2005 10e 182 16 15 1 75 MK Epinotia_tetraquetrana_(Haworth,_1811) 23
2005 10e 182 16 15 1 75 MK Apotomis_sororculana_(Zetterstedt,_1839) 1
2005 10e 182 16 15 1 75 MK Glyphipterix_haworthana_(Stephens,_1834) 1
2005 10e 182 16 15 1 75 MK Apotomis_moestana_(Wocke,_1862) 1
2005 10e 182 16 15 1 75 MK Phiaris_bipunctana_(Fabricius,_1794) 1
2005 10e 182 16 15 1 75 MK Apotomis_algidana_Krogerus,_1946 1
2005 10e 182 16 15 1 75 MK Argyroploce_lediana_(Linnaeus,_1758) 1

2005 10e 182 16 15 1 75 MK Sympistis_heliophila_(Paykull,_1793) 1
2005 10e 182 16 15 1 75 MK Pammene_clanculana_(Tengström,_1869) 1
2005 10e 182 16 15 1 75 MK Choristoneura_albaniana_(Walker,_1863) 1
2005 10e 182 16 15 1 75 MK Prolita_sexpunctella_(Fabricius,_1794) 4
2005 10e 182 16 15 1 75 VZ Gesneria_centuriella_(Denis_&_Schiffermüller,_1775) 1
2005 10e 182 16 15 1 75 VZ Phiaris_bipunctana_(Fabricius,_1794) 1
2005 10e 182 16 15 1 75 VZ Boloria_frigga_(Thunberg,_1791) 1
2005 10e 182 16 15 1 75 VZ Hedya_atropunctana_(Zetterstedt,_1839) 2
2005 10e 182 16 15 1 75 VZ Boloria_aquilonaris_(Stichel,_1908) 1
2005 10e 182 16 15 1 75 VZ Lozotaenia_forsterana_(Fabricius,_1781) 3
2005 10e 182 16 15 1 75 VZ Eulia_ministrana_(Linnaeus,_1758) 1
2005 10e 182 16 15 1 75 VZ Phiaris_septentrionana_(Curtis,_1835) 1
2005 10e 182 16 15 1 75 VZ Epinotia_tetraquetrana_(Haworth,_1811) 9
2005 10e 182 16 15 1 75 VZ Pammene_clanculana_(Tengström,_1869) 2
2005 10e 182 16 15 1 75 VZ Micropterix_aureatella_(Scopoli,_1763) 1
2005 10e 182 16 15 1 75 VZ Glyphipterix_haworthana_(Stephens,_1834) 1
2005 10e 182 16 15 1 75 VZ Paraswammerdamia_conspersella_(Tengström,_1848) 1
2005 10e 182 16 15 1 75 VZ Gypsonoma_nitidulana_(Lienig_&_Zeller,_1846) 1
2005 10e 182 16 15 1 75 VZ Rheumaptera_subhastata_(Nolcken,_1870) 1
2005 4ne 182 16 13 1 100 MK Epinotia_tetraquetrana_(Haworth,_1811) 24
2005 4ne 182 16 13 1 100 MK Ancylis_myrtillana_(Treitschke,_1830) 1
2005 4ne 182 16 13 1 100 MK Argyroploce_lediana_(Linnaeus,_1758) 1
2005 4ne 182 16 13 1 100 MK Syndemis_musculana_(Hübner,_1799) 1
2005 4ne 182 16 13 1 100 MK Plutella_xylostella_(Linnaeus,_1758) 1
2005 4ne 182 16 13 1 100 MK Ancylis_unguicella_(Linnaeus,_1758) 2
2005 4ne 182 16 13 1 100 MK Hedya_atropunctana_(Zetterstedt,_1839) 1
2005 4ne 182 16 13 1 100 MK Paraswammerdamia_conspersella_(Tengström,_1848) 3
2005 4ne 182 16 13 1 100 MK Parornix_loganella_(Stainton,_1848) 1
2005 4ne 182 16 13 1 100 MK Ancylis_uncella_(Denis_&_Schiffermüller,_1775) 1
2005 4ne 182 16 13 1 100 MK Neofaculta_infernella_(Herrich-Schäffer,_1854) 1
2005 4ne 182 16 13 1 100 MK Prolita_sexpunctella_(Fabricius,_1794) 3
2005 4ne 182 16 13 1 100 VZ Epinotia_tetraquetrana_(Haworth,_1811) 15
2005 4ne 182 16 13 1 100 VZ Ancylis_unguicella_(Linnaeus,_1758) 2
2005 4ne 182 16 13 1 100 VZ Gypsonoma_nitidulana_(Lienig_&_Zeller,_1846) 2
2005 4ne 182 16 13 1 100 VZ Plutella_xylostella_(Linnaeus,_1758) 1
2005 4ne 182 16 13 1 100 VZ Parornix_loganella_(Stainton,_1848) 1
2005 4ne 182 16 13 1 100 VZ Prolita_sexpunctella_(Fabricius,_1794) 1
2005 4ne 182 16 13 1 100 VZ Syndemis_musculana_(Hübner,_1799) 1
2005 0.5ne 182 14 13 1 75 MK Hedya_atropunctana_(Zetterstedt,_1839) 6
2005 0.5ne 182 14 13 1 75 MK Epinotia_tetraquetrana_(Haworth,_1811) 13
2005 0.5ne 182 14 13 1 75 MK Pammene_clanculana_(Tengström,_1869) 1
2005 0.5ne 182 14 13 1 75 MK Apotomis_sororculana_(Zetterstedt,_1839) 1
2005 0.5ne 182 14 13 1 75 VZ Hedya_atropunctana_(Zetterstedt,_1839) 1
2005 0.5ne 182 14 13 1 75 VZ Epinotia_tetraquetrana_(Haworth,_1811) 13
2005 0.5ne 182 14 13 1 75 VZ Syndemis_musculana_(Hübner,_1799) 1
2005 0.5ne 182 14 13 1 75 VZ Parornix_loganella_(Stainton,_1848) 1
2005 0.5ne 182 14 13 1 75 VZ Phyllonorycter_hilarella_(Zetterstedt,_1839) 1
2005 0.5ne 182 14 13 1 75 VZ Phyllonorycter_rolandi_(Svensson,_1966) 1
2005 1.4e 181 18 12 1 100 MK Epinotia_tetraquetrana_(Haworth,_1811) 44
2005 1.4e 181 18 12 1 100 MK Choristoneura_albaniana_(Walker,_1863) 2
2005 1.4e 181 18 12 1 100 MK Ancylis_uncella_(Denis_&_Schiffermüller,_1775) 1
2005 1.4e 181 18 12 1 100 MK Hedya_atropunctana_(Zetterstedt,_1839) 1
2005 1.4e 181 18 12 1 100 MK Swammerdamia_caesiella_(Hübner,_1796) 2
2005 1.4e 181 18 12 1 100 MK Apotomis_sororculana_(Zetterstedt,_1839) 1
2005 1.4e 181 18 12 1 100 MK Stictea_mygindiana_(Denis_&_Schiffermüller,_1775) 1
2005 1.4e 181 20 12 1 100 VZ Falcaria_lacertinaria_(Linnaeus,_1758) 1
2005 1.4e 181 20 12 1 100 VZ Choristoneura_albaniana_(Walker,_1863) 1
2005 1.4e 181 20 12 1 100 VZ Epinotia_tetraquetrana_(Haworth,_1811) 45
2005 1.4e 181 20 12 1 100 VZ Ancylis_myrtillana_(Treitschke,_1830) 1
2005 1.4e 181 20 12 1 100 VZ Syndemis_musculana_(Hübner,_1799) 3
2005 1.4e 181 20 12 1 100 VZ Pammene_clanculana_(Tengström,_1869) 1
2005 3sw 181 20 13 1 100 MK Ancylis_myrtillana_(Treitschke,_1830) 9
2005 3sw 181 20 13 1 100 MK Hedya_atropunctana_(Zetterstedt,_1839) 2
2005 3sw 181 20 13 1 100 MK Udea_inquinatalis_(Lienig_&_Zeller,_1846) 1
2005 3sw 181 20 13 1 100 MK Ancylis_unguicella_(Linnaeus,_1758) 1
2005 3sw 181 20 13 1 100 MK Mompha_raschkiella_(Zeller,_1838) 1
2005 3sw 181 20 13 1 100 MK Argyroploce_lediana_(Linnaeus,_1758) 1
2005 3sw 181 20 13 1 100 MK Elachista_diederichsiella_E._Hering,_1889 1
2005 3sw 181 20 13 1 100 MK Epinotia_tetraquetrana_(Haworth,_1811) 10
2005 3sw 181 20 13 1 100 MK Prolita_sexpunctella_(Fabricius,_1794) 2
2005 3sw 181 20 13 1 100 MK Nematopogon_pilella_(Denis_&_Schiffermüller,_1775) 1

2005 3sw 181 20 13 1 100 MK Swammerdamia_caesiella_(Hübner,_1796) 1
2005 3sw 181 20 13 1 100 MK Monopis_spilotella_Tengström,_1848 1
2005 3sw 181 22 13 1 100 VZ Ancylis_myrtillana_(Treitschke,_1830) 4
2005 3sw 181 22 13 1 100 VZ Apotomis_sororculana_(Zetterstedt,_1839) 1
2005 3sw 181 22 13 1 100 VZ Epinotia_tetraquetrana_(Haworth,_1811) 15
2005 3sw 181 22 13 1 100 VZ Prolita_sexpunctella_(Fabricius,_1794) 2
2005 3sw 181 22 13 1 100 VZ Syndemis_musculana_(Hübner,_1799) 2
2005 3sw 181 22 13 1 100 VZ Stictea_mygindiana_(Denis_&_Schiffermüller,_1775) 1
2005 3sw 181 22 13 1 100 VZ Ancylis_unguicella_(Linnaeus,_1758) 3
2005 3sw 181 22 13 1 100 VZ Argyroploce_lediana_(Linnaeus,_1758) 1
2005 3sw 181 22 13 1 100 VZ Gypsonoma_nitidulana_(Lienig_&_Zeller,_1846) 1
2005 3sw 181 22 13 1 100 VZ Ancylis_uncella_(Denis_&_Schiffermüller,_1775) 1
2005 3sw 181 22 13 1 100 VZ Elophos_vittaria_(Thunberg,_1788) 1
2005 10sw 181 20 12 1 100 MK Eulia_ministrana_(Linnaeus,_1758) 4
2005 10sw 181 20 12 1 100 MK Apotomis_sororculana_(Zetterstedt,_1839) 1
2005 10sw 181 20 12 1 100 MK Ematurga_atomaria_(Linnaeus,_1758) 2
2005 10sw 181 20 12 1 100 MK Carpatolechia_epomidella_(Tengström,_1869) 1
2005 10sw 181 20 12 1 100 MK Lozotaenia_forsterana_(Fabricius,_1781) 1
2005 10sw 181 20 12 1 100 MK Neofaculta_infernella_(Herrich-Schäffer,_1854) 2
2005 10sw 181 20 12 1 100 MK Ancylis_unguicella_(Linnaeus,_1758) 1
2005 10sw 181 20 12 1 100 MK Prolita_sexpunctella_(Fabricius,_1794) 3
2005 10sw 181 20 12 1 100 MK Ancylis_myrtillana_(Treitschke,_1830) 1
2005 10sw 181 20 12 1 100 MK Epinotia_tetraquetrana_(Haworth,_1811) 5
2005 10sw 181 20 12 1 100 MK Syndemis_musculana_(Hübner,_1799) 2
2005 10sw 181 20 12 1 100 MK Micropterix_aureatella_(Scopoli,_1763) 2
2005 10sw 181 20 12 1 100 MK Eupithecia_satyrata_(Hübner,_1813) 1
2005 10sw 181 20 12 1 100 MK Eupithecia_gelidata_Möschler,_1860 1
2005 10sw 181 20 12 1 100 VZ Eulia_ministrana_(Linnaeus,_1758) 8
2005 10sw 181 20 12 1 100 VZ Ematurga_atomaria_(Linnaeus,_1758) 1
2005 10sw 181 20 12 1 100 VZ Lozotaenia_forsterana_(Fabricius,_1781) 2
2005 10sw 181 20 12 1 100 VZ Prolita_sexpunctella_(Fabricius,_1794) 2
2005 10sw 181 20 12 1 100 VZ Ancylis_myrtillana_(Treitschke,_1830) 4
2005 10sw 181 20 12 1 100 VZ Epinotia_tetraquetrana_(Haworth,_1811) 10
2005 10sw 181 20 12 1 100 VZ Elophos_vittaria_(Thunberg,_1788) 1
2005 10sw 181 20 12 1 100 VZ Eupithecia_satyrata_(Hübner,_1813) 1
2005 10sw 181 20 12 1 100 VZ Eupithecia_virgaureata_Doubleday,_1861 1
2005 10sw 181 20 12 1 100 VZ Paraswammerdamia_conspersella_(Tengström,_1848) 2
2005 10sw 181 20 12 1 100 VZ Neofaculta_infernella_(Herrich-Schäffer,_1854) 2
2005 10sw 181 20 12 1 100 VZ Chionodes_viduella_(Fabricius,_1794) 1
2005 17s 180 20 15 1 100 MK Ancylis_myrtillana_(Treitschke,_1830) 8
2005 17s 180 20 15 1 100 MK Eupithecia_intricata_(Zetterstedt,_1839) 1
2005 17s 180 20 15 1 100 MK Lozotaenia_forsterana_(Fabricius,_1781) 9
2005 17s 180 20 15 1 100 MK Eupithecia_satyrata_(Hübner,_1813) 1
2005 17s 180 20 15 1 100 MK Syndemis_musculana_(Hübner,_1799) 1
2005 17s 180 20 15 1 100 MK Phiaris_septentrionana_(Curtis,_1835) 1
2005 17s 180 20 15 1 100 MK Ancylis_unguicella_(Linnaeus,_1758) 1
2005 17s 180 20 15 1 100 MK Udea_decrepitalis_(Herrich-Schäffer,_1848) 1
2005 17s 180 20 15 1 100 MK Epinotia_tetraquetrana_(Haworth,_1811) 35
2005 17s 180 20 15 1 100 MK Elachista_exactella_(Herrich-Schäffer,_1855) 1
2005 17s 180 20 15 1 100 MK Hedya_atropunctana_(Zetterstedt,_1839) 5
2005 17s 180 20 15 1 100 VZ Erebia_pandrose_(Borkhausen,_1788) 1
2005 17s 180 20 15 1 100 VZ Hedya_atropunctana_(Zetterstedt,_1839) 2
2005 17s 180 20 15 1 100 VZ Glacies_coracina_(Esper,_1805) 1
2005 17s 180 20 15 1 100 VZ Ancylis_myrtillana_(Treitschke,_1830) 2
2005 17s 180 20 15 1 100 VZ Macaria_carbonaria_(Clerck,_1759) 1
2005 17s 180 20 15 1 100 VZ Epinotia_tetraquetrana_(Haworth,_1811) 47
2005 17s 180 20 15 1 100 VZ Ematurga_atomaria_(Linnaeus,_1758) 2
2005 17s 180 20 15 1 100 VZ Syndemis_musculana_(Hübner,_1799) 4
2005 17s 180 20 15 1 100 VZ Lozotaenia_forsterana_(Fabricius,_1781) 2
2005 17s 180 20 15 1 100 VZ Elophos_vittaria_(Thunberg,_1788) 1
2005 17s 180 20 15 1 100 VZ Choristoneura_albaniana_(Walker,_1863) 2
2005 17s 180 20 15 1 100 VZ Xanthorhoe_spadicearia_(Denis_&_Schiffermüller,_1775) 1
2005 17s 180 20 15 1 100 VZ Glyphipterix_haworthana_(Stephens,_1834) 1
2005 38s 180 18 14 1 50 MK Boloria_euphrosyne_(Linnaeus,_1758) 1
2005 38s 180 18 14 1 50 MK Ancylis_myrtillana_(Treitschke,_1830) 3
2005 38s 180 18 14 1 50 MK Udea_inquinatalis_(Lienig_&_Zeller,_1846) 1
2005 38s 180 18 14 1 50 MK Phiaris_schulziana_(Fabricius,_1776) 1
2005 38s 180 18 14 1 50 MK Clepsis_senecionana_(Hübner,_1819) 2
2005 38s 180 18 14 1 50 MK Epinotia_tetraquetrana_(Haworth,_1811) 42
2005 38s 180 18 14 1 50 MK Choristoneura_albaniana_(Walker,_1863) 3
2005 38s 180 18 14 1 50 MK Paraswammerdamia_conspersella_(Tengström,_1848) 1

2005 38s 180 18 14 1 50 MK Apotomis_fraterculana_Krogerus,_1946 1
2005 38s 180 18 14 1 50 VZ Boloria_euphrosyne_(Linnaeus,_1758) 2
2005 38s 180 18 14 1 50 VZ Ancylis_myrtillana_(Treitschke,_1830) 4
2005 38s 180 18 14 1 50 VZ Udea_inquinatalis_(Lienig_&_Zeller,_1846) 1
2005 38s 180 18 14 1 50 VZ Phiaris_schulziana_(Fabricius,_1776) 2
2005 38s 180 18 14 1 50 VZ Ancylis_unguicella_(Linnaeus,_1758) 1
2005 38s 180 18 14 1 50 VZ Epinotia_tetraquetrana_(Haworth,_1811) 45
2005 38s 180 18 14 1 50 VZ Choristoneura_albaniana_(Walker,_1863) 3
2005 38s 180 18 14 1 50 VZ Syndemis_musculana_(Hübner,_1799) 1
2005 38s 180 18 14 1 50 VZ Hedya_atropunctana_(Zetterstedt,_1839) 1
2005 38s 180 18 14 1 50 VZ Chionodes_viduella_(Fabricius,_1794) 1
2007 3sw 185 20 20 1 0 mk Gesneria_centuriella_(Denis_&_Schiffermüller,_1775) 2
2007 3sw 185 20 20 1 0 mk Ematurga_atomaria_(Linnaeus,_1758) 1
2007 3sw 185 20 20 1 0 mk Plutella_xylostella_(Linnaeus,_1758) 1
2007 3sw 185 20 20 1 0 mk Epinotia_tetraquetrana_(Haworth,_1811) 7
2007 3sw 185 20 20 1 0 mk Argyroploce_lediana_(Linnaeus,_1758) 2
2007 3sw 185 20 20 1 0 mk Anania_funebris_(Ström,_1768) 2
2007 3sw 185 20 20 1 0 mk Ancylis_unguicella_(Linnaeus,_1758) 3
2007 3sw 185 20 20 1 0 mk Elachista_nielswolffi_Svensson,_1976 1
2007 3sw 185 20 20 1 0 mk Coleophora_vitisella_Gregson,_1856 1
2007 3sw 185 20 20 1 0 mk Phiaris_dissolutana_(Stange,_1866) 1
2007 3sw 185 20 20 1 0 mk Prolita_sexpunctella_(Fabricius,_1794) 1
2007 3sw 185 20 20 1 0 mk Parornix_loganella_(Stainton,_1848) 1
2007 3sw 185 20 20 1 0 vz Gesneria_centuriella_(Denis_&_Schiffermüller,_1775) 1
2007 3sw 185 20 20 1 0 vz Plutella_xylostella_(Linnaeus,_1758) 2
2007 3sw 185 20 20 1 0 vz Epinotia_tetraquetrana_(Haworth,_1811) 5
2007 3sw 185 20 20 1 0 vz Argyroploce_lediana_(Linnaeus,_1758) 2
2007 3sw 185 20 20 1 0 vz Mompha_raschkiella_(Zeller,_1838) 1
2007 3sw 185 20 20 1 0 vz Prolita_sexpunctella_(Fabricius,_1794) 2
2007 3sw 185 20 20 1 0 vz Gypsonoma_nitidulana_(Lienig_&_Zeller,_1846) 1
2007 3sw 185 20 20 1 0 vz Phiaris_obsoletana_(Zetterstedt,_1839) 1
2007 1.4e 185 22 20 0 0 mk Plutella_xylostella_(Linnaeus,_1758) 3
2007 1.4e 185 22 20 0 0 mk Epinotia_tetraquetrana_(Haworth,_1811) 6
2007 1.4e 185 22 20 0 0 mk Apotomis_moestana_(Wocke,_1862) 1
2007 1.4e 185 22 20 0 0 vz Hedya_atropunctana_(Zetterstedt,_1839) 4
2007 1.4e 185 22 20 0 0 vz Epinotia_tetraquetrana_(Haworth,_1811) 5
2007 1.4e 185 22 20 0 0 vz Ancylis_comptana_(Frölich,_1828) 1
2007 1.4e 185 22 20 0 0 vz Syndemis_musculana_(Hübner,_1799) 1
2007 1.4e 185 22 20 0 0 vz Ancylis_uncella_(Denis_&_Schiffermüller,_1775) 1
2007 10sw 185 20 20 1 1 mk Eulia_ministrana_(Linnaeus,_1758) 1
2007 10sw 185 20 20 1 1 mk Plutella_xylostella_(Linnaeus,_1758) 4
2007 10sw 185 20 20 1 1 mk Phiaris_schulziana_(Fabricius,_1776) 1
2007 10sw 185 20 20 1 1 mk Micropterix_aureatella_(Scopoli,_1763) 2
2007 10sw 185 20 20 1 1 mk Xanthorhoe_spadicearia_(Denis_&_Schiffermüller,_1775) 1
2007 10sw 185 20 20 1 1 mk Chionodes_viduella_(Fabricius,_1794) 1
2007 10sw 185 20 20 1 1 mk Prolita_sexpunctella_(Fabricius,_1794) 3
2007 10sw 185 20 20 1 1 mk Ancylis_unguicella_(Linnaeus,_1758) 1
2007 10sw 185 20 20 1 1 vz Stictea_mygindiana_(Denis_&_Schiffermüller,_1775) 1
2007 10sw 185 20 20 1 1 vz Ematurga_atomaria_(Linnaeus,_1758) 1
2007 10sw 185 20 20 1 1 vz Phiaris_schulziana_(Fabricius,_1776) 1
2007 10sw 185 20 20 1 1 vz Micropterix_aureatella_(Scopoli,_1763) 1
2007 10sw 185 20 20 1 1 vz Argyroploce_lediana_(Linnaeus,_1758) 1
2007 10sw 185 20 20 1 1 vz Glyphipterix_haworthana_(Stephens,_1834) 3
2007 10sw 185 20 20 1 1 vz Plutella_xylostella_(Linnaeus,_1758) 1
2007 10sw 185 20 20 1 1 vz Arctia_lapponica_(Thunberg,_1791) 1
2007 10sw 185 20 20 1 1 vz Chionodes_viduella_(Fabricius,_1794) 1
2007 10sw 185 20 20 1 1 vz Ancylis_unguicella_(Linnaeus,_1758) 4
2007 10sw 185 20 20 1 1 vz Coleophora_vitisella_Gregson,_1856 1
2007 10sw 185 20 20 1 1 vz Elachista_kilmunella_Stainton,_1849 1
2007 17s 185 18 20 0 1 mk Boloria_euphrosyne_(Linnaeus,_1758) 1
2007 17s 185 18 20 0 1 mk Lozotaenia_forsterana_(Fabricius,_1781) 1
2007 17s 185 18 20 0 1 mk Eulia_ministrana_(Linnaeus,_1758) 1
2007 17s 185 18 20 0 1 mk Rheumaptera_subhastata_(Nolcken,_1870) 1
2007 17s 185 18 20 0 1 mk Phiaris_turfosana_(Herrich-Schäffer,_1851) 2
2007 17s 185 18 20 0 1 mk Phiaris_schulziana_(Fabricius,_1776) 4
2007 17s 185 18 20 0 1 mk Micropterix_aureatella_(Scopoli,_1763) 2
2007 17s 185 18 20 0 1 mk Epinotia_tetraquetrana_(Haworth,_1811) 5
2007 17s 185 18 20 0 1 mk Glacies_coracina_(Esper,_1805) 2
2007 17s 185 18 20 0 1 mk Coleophora_frischella_(Linnaeus,_1758) 1
2007 17s 185 18 20 0 1 mk Ancylis_unguicella_(Linnaeus,_1758) 1
2007 17s 185 18 20 0 1 mk Prolita_sexpunctella_(Fabricius,_1794) 1

2007 17s 185 18 20 0 1 mk Udea_inquinatalis_(Lienig_&_Zeller,_1846) 1
2007 17s 185 18 20 0 1 vz Boloria_euphrosyne_(Linnaeus,_1758) 1
2007 17s 185 18 20 0 1 vz Lozotaenia_forsterana_(Fabricius,_1781) 1
2007 17s 185 18 20 0 1 vz Rheumaptera_subhastata_(Nolcken,_1870) 1
2007 17s 185 18 20 0 1 vz Ematurga_atomaria_(Linnaeus,_1758) 1
2007 17s 185 18 20 0 1 vz Phiaris_turfosana_(Herrich-Schäffer,_1851) 1
2007 17s 185 18 20 0 1 vz Micropterix_aureatella_(Scopoli,_1763) 2
2007 17s 185 18 20 0 1 vz Epinotia_tetraquetrana_(Haworth,_1811) 3
2007 17s 185 18 20 0 1 vz Pediasia_truncatellus_(Zetterstedt,_1839) 1
2007 17s 185 18 20 0 1 vz Syndemis_musculana_(Hübner,_1799) 1
2007 17s 185 18 20 0 1 vz Prolita_sexpunctella_(Fabricius,_1794) 2
2007 17s 185 18 20 0 1 vz Eupithecia_satyrata_(Hübner,_1813) 1
2007 38s 185 18 20 0 0 mk Boloria_euphrosyne_(Linnaeus,_1758) 2
2007 38s 185 18 20 0 0 mk Ancylis_myrtillana_(Treitschke,_1830) 1
2007 38s 185 18 20 0 0 mk Epinotia_tetraquetrana_(Haworth,_1811) 3
2007 38s 185 18 20 0 0 mk Phiaris_schulziana_(Fabricius,_1776) 2
2007 38s 185 18 20 0 0 mk Phiaris_turfosana_(Herrich-Schäffer,_1851) 1
2007 38s 185 18 20 0 0 mk Ancylis_unguicella_(Linnaeus,_1758) 4
2007 38s 185 18 20 0 0 mk Cyclophora_albipunctata_(Hufnagel,_1767) 1
2007 38s 185 18 20 0 0 mk Chionodes_viduella_(Fabricius,_1794) 1
2007 38s 185 18 20 0 0 mk Prolita_sexpunctella_(Fabricius,_1794) 1
2007 38s 185 18 20 0 0 mk Pammene_clanculana_(Tengström,_1869) 1
2007 38s 185 18 20 0 0 vz Boloria_eunomia_(Esper,_1800) 2
2007 38s 185 18 20 0 0 vz Eulia_ministrana_(Linnaeus,_1758) 1
2007 38s 185 18 20 0 0 vz Agriades_optilete_(Knoch,_1781) 1
2007 38s 185 18 20 0 0 vz Phiaris_turfosana_(Herrich-Schäffer,_1851) 2
2007 38s 185 18 20 0 0 vz Epinotia_tetraquetrana_(Haworth,_1811) 2
2007 38s 185 18 20 0 0 vz Argyroploce_lediana_(Linnaeus,_1758) 1
2007 38s 185 18 20 0 0 vz Apotomis_lemniscatana_(Kennel,_1901) 1
2008 45e 211 14 12 1 50 mk Agriades_optilete_(Knoch,_1781) 2
2008 45e 211 14 12 1 50 mk Phiaris_obsoletana_(Zetterstedt,_1839) 1
2008 45e 211 14 12 1 50 mk Sparganothis_rubicundana_(Herrich-Schäffer,_1856) 1
2008 45e 211 14 12 1 50 mk Macaria_fusca_(Thunberg,_1792) 2
2008 45e 211 14 12 1 50 VZ Agriades_optilete_(Knoch,_1781) 3
2008 45e 211 14 12 1 50 vz Entephria_caesiata_(Denis_&_Schiffermüller,_1775) 1
2008 45e 211 14 12 1 50 vz Sympistis_heliophila_(Paykull,_1793) 1
2008 45e 211 14 12 1 50 vz Macaria_fusca_(Thunberg,_1792) 1
2008 45e 211 14 12 1 50 vz Phiaris_schulziana_(Fabricius,_1776) 1
2008 45e 211 14 12 1 50 vz Phiaris_turfosana_(Herrich-Schäffer,_1851) 2
2008 28e 211 12 11 1 100 mk Macaria_fusca_(Thunberg,_1792) 2
2008 28e 211 12 11 1 100 mk Phiaris_obsoletana_(Zetterstedt,_1839) 1
2008 28e 211 12 11 1 100 vz Entephria_polata_(Duponchel,_1830) 1
2008 28e 211 12 11 1 100 vz Macaria_fusca_(Thunberg,_1792) 1
2008 28e 211 12 11 1 100 vz Phiaris_obsoletana_(Zetterstedt,_1839) 2
2008 10e 211 12 10 1 100 mk Taleporia_tubulosa_(Retzius,_1783) 1
2008 10e 211 12 10 1 100 mk Argyresthia_conjugella_Zeller,_1839 1
2008 10e 211 12 10 1 100 mk Epinotia_cruciana_(Linnaeus,_1761) 1
2008 10e 211 12 10 1 100 vz Macaria_brunneata_(Thunberg,_1784) 1
2008 4ne 211 12 11 1 75 mk Mompha_idaei_(Zeller,_1839) 1
2008 4ne 211 12 11 1 75 mk Epinotia_cruciana_(Linnaeus,_1761) 2
2008 4ne 211 12 11 1 75 mk Argyresthia_pygmaeella_(Denis_&_Schiffermüller,_1775) 13
2008 4ne 211 12 11 1 75 vz Epinotia_cruciana_(Linnaeus,_1761) 2
2008 4ne 211 12 11 1 75 vz Argyresthia_pygmaeella_(Denis_&_Schiffermüller,_1775) 1
2008 0.5ne 211 10 9 0 50 mk Mompha_idaei_(Zeller,_1839) 1
2008 0.5ne 211 10 9 0 50 mk Argyresthia_pygmaeella_(Denis_&_Schiffermüller,_1775) 4
2008 0.5ne 211 10 9 0 50 mk Phyllonorycter_hilarella_(Zetterstedt,_1839) 1
2008 0.5ne 211 10 9 0 50 vz Argyresthia_pygmaeella_(Denis_&_Schiffermüller,_1775) 3
2008 1.4e 211 10 9 0 50 mk Mompha_idaei_(Zeller,_1839) 1
2008 1.4e 211 10 9 0 50 vz Mompha_idaei_(Zeller,_1839) 1
2008 3sw 210 20 11 2 50 mk Argyresthia_pygmaeella_(Denis_&_Schiffermüller,_1775) 1
2008 3sw 210 20 11 2 50 vz . 0
2008 10sw 210 20 12 1 0 mk Eudonia_alpina_(Curtis,_1850) 1
2008 10sw 210 20 12 1 0 vz Entephria_caesiata_(Denis_&_Schiffermüller,_1775) 2
2008 17s 210 10 12 2 0 mk Agriades_optilete_(Knoch,_1781) 1
2008 17s 210 10 12 2 0 mk Xanthorhoe_abrasaria_(Herrich-Schäffer,_1855) 1
2008 17s 210 10 12 2 0 mk Scopula_ternata_(Schrank,_1802) 1
2008 17s 210 10 12 2 0 mk Coleophora_virgaureae_Stainton,_1857 1
2008 17s 210 10 12 2 0 mk Phiaris_turfosana_(Herrich-Schäffer,_1851) 2
2008 17s 210 10 12 2 0 mk Phiaris_obsoletana_(Zetterstedt,_1839) 1
2008 17s 210 10 12 2 0 mk Phiaris_schulziana_(Fabricius,_1776) 2
2008 17s 210 10 12 2 0 vz Agriades_optilete_(Knoch,_1781) 1

2008 17s 210 10 12 2 0 vz Xanthorhoe_abrasaria_(Herrich-Schäffer,_1855) 3
2008 17s 210 10 12 2 0 vz Phiaris_obsoletana_(Zetterstedt,_1839) 2
2008 38s 210 18 13 2 0 mk Hylaea_fasciaria_(Linnaeus,_1758) 1
2008 38s 210 18 13 2 0 mk Phiaris_schulziana_(Fabricius,_1776) 2
2008 38s 210 18 13 2 0 vz Dysstroma_infuscata_(Tengström,_1869) 1
2008 38s 210 18 13 2 0 vz Argyroploce_lediana_(Linnaeus,_1758) 1
